# Burden and trends of major depressive disorders among women of childbearing age and the impact of the COVID-19 pandemic: insights from the global burden of disease study 2021

**DOI:** 10.3389/fpsyt.2025.1630601

**Published:** 2025-09-18

**Authors:** Guobin Liao, Jiaoxue Wang, Qiaozhi Yu, Honglin Ma, Hezhong Yan, Zhaoyun He, Jun Tang

**Affiliations:** ^1^ Department of Gastroenterology, The 901 Hospital of Chinese People’s Liberation Army (PLA) Joint Service Support Unit, Hefei, China; ^2^ Department of Obstetrics and Gynecology, The 901 Hospital of Chinese People’s Liberation Army (PLA) Joint Service Support Unit, Hefei, China; ^3^ Department of Liver Disease, The Fifth Medical Center of Chinese People's Liberation Army (PLA) General Hospital, Beijing, China

**Keywords:** major depressive disorder, women of childbearing age, disease burden, COVID-19 pandemic, GBD 2021

## Abstract

**Background:**

Major depressive disorder (MDD) exhibits a pronounced female predominance, contributing substantially to disability-adjusted life-years (DALYs) among women of childbearing age (WCBA; 15–49 years). The COVID-19 pandemic intensified this burden via psychosocial stressors and disrupted healthcare access, yet integrated analyses of pre- and post-pandemic trends are scarce.

**Methods:**

Leveraging GBD 2021 data, we assessed MDD prevalence, incidence, and DALYs among WCBA globally, regionally, and nationally (1990–2021). Burden estimates were reported as point values with 95% uncertainty intervals (UIs). Temporal trends were quantified via estimated annual percentage change (EAPC) and absolute percentage change (PC). We employed autoregressive integrated moving average (ARIMA) models to compare pre-pandemic (1990–2019) and pandemic-inclusive (1990–2021) projections through 2036.

**Results:**

Global prevalent cases among WCBA surged from 49.6 million (95% UI: 41.6 to 60.2) in 1990 to 85.6 million (95% UI: 70.3 to 103.8) in 2021, reflecting a 25.7% acceleration during 2019–2021 versus 1.2% annual growth pre-pandemic. Prevalence rates declined marginally pre-2019 (EAPC: −0.38, 95% CI: −0.48 to −0.29) but reversed sharply post-pandemic (EAPC: 11.47, 95% CI: −0.56 to 24.95), reaching 4,394.55 per 100,000 population in 2021. Regionally, middle and low-middle socio-demographic index (SDI) regions accounted for over 55% of global cases (23 million [95% UI: 18.9 to 27.8] and 24.2 million [95% UI: 19.6 to 29.9], respectively) in 2021, while low SDI regions showed the fastest growth (160% since 1990). High-SDI regions exhibited extremes: the highest 2021 prevalence rate (5915.76 per 100,000 population) and steepest post-2019 surge (EAPC: 13.66). In 2021, the prevalence rates were highest in high-income North America (8403.17 per 100,000 population) and lowest in East Asia (1856.99 per 100,000 population). Nationally, India reported the highest prevalent cases (16.3 million, 19% of global share), while Greenland had the highest prevalence rate (13,822.85 per 100,000 population). Adolescents (15–19 years) experienced the largest pandemic-driven increase (30.06% PC), except in East Asia where prevalence rates declined (−11.53%). ARIMA projections suggest 103.06 million global prevalent cases by 2036—32% above pre-pandemic estimates—with high SDI regions persisting at 5,617.68 per 100,000 population.

**Conclusions:**

Our analysis reveals a dual crisis: high SDI regions face entrenched high prevalence rates (5,617.68 per 100,000 population projected), while low SDI regions carry substantial burden (4,593.77 per 100,000) with rapid case expansion (160% since 1990). The pandemic disproportionately impacted adolescents globally (+30.06% PC), yet East Asia demonstrated resilience (−11.53% PC). These findings demand stratified interventions: digital mental health tools in high-income settings, community-based screening in resource-limited areas, and adolescent-focused programs worldwide. Immediate policy action is needed to avert intergenerational mental health consequences.

## Introduction

Depression—a leading global cause of DALYs among mental disorders (1)—is characterized by persistent low mood and anhedonia. It is categorized into two subtypes: major depressive disorder (MDD) and dysthymia. MDD is an episodic mood disorder with shorter duration but more severe symptoms than dysthymia. In 2019, over 274 million people suffered from MDD worldwide (2). Alarmingly, women face twice the lifetime MDD risk of men (1, 3), with vulnerability peaking occurring during reproductive transitions—notably adolescence, peripartum, and perimenopause (4–7). This sex-specific susceptibility stems from dynamic interactions between ovarian hormone fluctuations and gendered psychosocial stressors (4). These suggest that the female reproductive cycle constitutes a unique biological vulnerability window for MDD. Therefore, it’s essential to consider the effects of childbearing stages and hormonal fluctuations when evaluating female patients (8). Critically, 25% of women with MDD report pre-pregnancy symptom onset (9). Untreated antenatal depression heightens risks of preterm birth, low birth weight, stillbirth, and maternal complications (e.g., perinatal morbidity, operative delivery, postpartum depression) (6, 10–13). These adverse outcomes may stem from hormonal disruptions, maternal stress, or reduced prenatal care adherence (14, 15). Furthermore, parents have MDD are also predisposing offspring to neurodevelopmental disorders (16) through gene–environment interaction, neural behavior circuits and social learning (17–19). These suggest that the consequences of MDD are intergenerational. Consequently, clinical practice guidelines advocate targeted screening for MDD in women during pregnancy (20).

The outbreak of the COVID-19 pandemic has further compounded mental health challenges worldwide, particularly for MDD (21). The pandemic introduced unprecedented disruptions to daily life, including lockdowns, economic instability, reduced access to healthcare services, increased caregiving responsibilities, and economic uncertainty, which have led many individuals to experience unprecedented levels of depression and stress (22). Studies have shown a marked increase in prevalence of MDD during the pandemic; for example, MDD prevalence increased by 28% globally in 2020 (23). Furthermore, the greater increase in prevalence among females compared males has resulted in an even larger sex disparity than before the pandemic (23). For women of childbearing age (WCBA), pandemic-specific stressors—including school closures (increasing childcare demands), remote work conflicts, and reduced access to reproductive healthcare—exacerbated pre-existing vulnerabilities tied to hormonal cycles and gendered caregiving roles (24–26).

Despite its increasing burden, comprehensive data on regional and longitudinal trends in MDD among WCBA. Moreover, how the ongoing COVID-19 pandemic shapes the burden of MDD among WCBA remains unclear. Therefore, a comprehensive analysis of the disease status and trends of MDD among WCBA is needed. Using the latest GBD 2021 data, we analyzed MDD incidence, prevalence, and DALYs among WCBA at the global, regional, and national levels from 1990 to 2021. We compared burden distribution and changes across age groups, emphasized the impact of the COVID-19 pandemic, and projected future prevalence trends through 2036. By integrating pre- and post-pandemic data into ARIMA models, we aimed to observe the long-term impact of a pandemic, providing actionable insights for post-pandemic health policy for MDD among WCBA.

## Methods

### Data sources

We analyzed the GBD 2021 database (1), which provides epidemiological estimates for 371 diseases/injuries across 204 countries and 21 regions (1990–2021). MDD was defined using the Diagnostic and Statistical Manual of Mental Disorders, 4th Edition (DSM-IV) and International Classification of Diseases, 10th Revision (ICD-10) criteria. Cases attributable to medical conditions or substance use were excluded to focus on primary depressive epidemiology (23). Data were extracted through the GHDx platform [http://ghdx.healthdata.org/gbd-results-tool], with parameters customized to filter WCBA-specific metrics (prevalence, incidence, DALYs). Ethical approval was waived as the study relied exclusively on de-identified, publicly accessible GBD data, adhering to institutional guidelines for secondary data analysis.

### Socio-demographic index

The socio-demographic index (SDI) was introduced by the Institute for Health Metrics and Evaluation (IHME) in 2015. It’s a comprehensive indicator designed to assess the development level of countries or regions. This study leverages SDI to contextualize how socioeconomic disparities influence MDD burden trajectories among WCBA. In short, the SDI aggregates three normalized indicators (0–1 scale): fertility rate among individuals <25 years, mean educational attainment for those ≥15 years, and lag-distributed income per capita. These components were synthesized via geometric mean to balance their contributions to socioeconomic development. SDI values were scaled to 0–100 (0: lowest income, least education, highest fertility; 100: highest income, most education, lowest fertility) to enhance interpretability. For this analysis, countries were stratified into five SDI quintiles based on 2021 values: low (0–0.45), low-middle (0.45–0.61), middle (0.61–0.69), high-middle (0.69–0.81), and high (0.81–1) (27). This stratification aligns with GBD conventions but focuses on WCBA-specific vulnerability patterns.

### Estimated annual percentage change and percentage change

To analyze dynamic trends in MDD burden among WCBA, we used the EAPC—a regression-based metric capturing annualized growth rates—to assess both pre-pandemic (1990–2019) and pandemic-era (2019–2021) trends. In prior research, it has been comprehensively utilized to monitor trends in indicators such as prevalence and incidence rates across particular time intervals (28). Statistical assumptions for EAPC calculation: a log-linear relationship between disease rates and time; normally distributed errors in the linear regression model; and independence of observations across years. Given the observed significant fluctuations during 2019–2021, we employed two complementary strategies: calculated separate EAPCs for pre-pandemic (1990–2019) and pandemic (2019–2021) periods; and reported absolute percentage change (PC) for short-term disruptions. This study is designed to estimate the dynamic trends in the prevalence, incidence, and DALYs of MDD among WCBA from 1990 to 2019 and 2019 to 2021. EAPC was derived from a linear regression of log-transformed rates (*y* = *α* + *β*x + *ϵ*), where *β* represents the annualized rate of change (*EAPC* = 100 × (*exp*(*β*) − 1)). The calculation of EAPC is grounded in the process of fitting the natural logarithm of the rate within a regression model. Here, time serves as a variable, and the natural logarithm of each observation is fitted into a straight-line function. Subsequently, the EAPC is computed based on the slope of this fitted line. In the context of the model, x represents the time variable in years, *y* denotes the natural logarithm of rates. The intercept is denoted as *α*, the slope as *β*, and *ϵ* represents the random error term. The 95% confidence intervals (CIs) for the EAPC are obtained from this fitted model. The interpretation of trend results is grounded in the 95% CIs. Trend significance was determined by 95% CIs: upward (CI lower limit >0), downward (CI upper limit <0), or stable (CI includes 0). To evaluate abrupt pandemic-related shifts, percentage change (PC) was computed between 2019 (pre-pandemic) and 2021 (post-pandemic), contrasting these with pre-2019 trends. The formula is: PC = ((Y_end_-Y_start_)/Y_start_) × 100%, where Y_end_ and Y_start_ are the rates at the end and start of the period, respectively.

### Model prediction

To assess the pandemic’s impact on future MDD burden, we applied the ARIMA (29) model—a time series method combining autoregressive (AR), differencing (I), and moving average (MA) components—to forecast prevalence trends among WCBA under two scenarios: Baseline projections (1990–2019 data, pre-pandemic), and post-pandemic adjustments (1990–2021 data, incorporating pandemic-era shifts). This enabled direct comparison of pandemic-driven deviations from historical patterns. In the ARIMA (p, d, q) model, the parameter “p” denotes the count of autoregressive terms, “d” represents the degree of differencing, and “q” indicates the number of moving average terms. ​Initial p, q ranges were identified via ACF/PACF plots, with final parameters selected by AIC/BIC minimization across a grid of combinations. For efficiency, parameter optimization was implemented via auto.arima(), which automates the grid search over (p, d, q) combinations under AIC/BIC constraints. Model optimization involved four steps: 1. Stationarity: achieved via differencing (d) and confirmed by KPSS tests. 2. residual normality: validated using Q-Q plots. 3. Model selection: AIC/BIC criteria identified the optimal model (lowest values). 4. Residual robustness: Ljung-Box tests confirmed residuals approximated white noise (p > 0.05).

In this study, data cleaning, computational processes, graph plotting and statistical analysis were conducted by R software (version 4.4.2). Visualizations were generated through the ggplot2 package. *P*-value < 0.05 was considered statistically significant.

## Results

### Global trends

**Figure 1:**
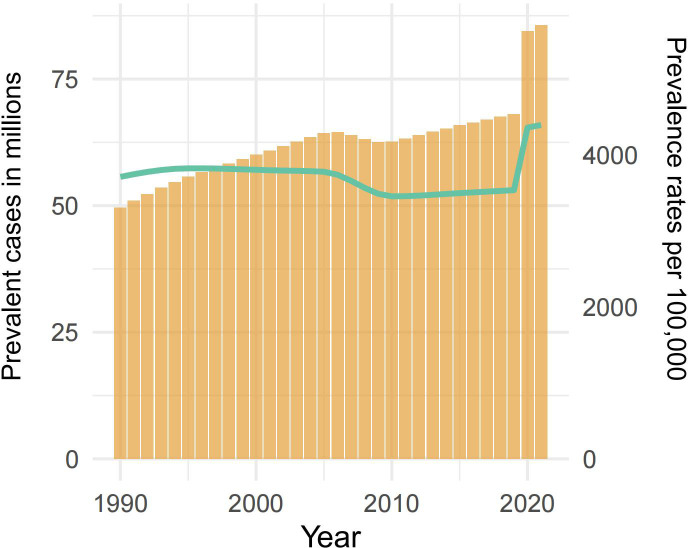
The prevalence of MDD among WCBA from 1990 to 2021. The bar graph shows the prevalent case (in millions) from 1990 to 2021 on the left-hand y-axis, while the line graph represents the prevalence rates per 100,000 population over the same period on the right-hand y-axis. WCBA, Women of Childbearing Age; MDD, major depressive disorders.

From 1990 to 2021, women of childbearing age (WCBA) experienced substantial increases in major depressive disorders (MDDs) burden globally. Prevalent cases increased gradually from 49.6 million (95% UI: 41.6 to 60.2) in 1990 to 68.1 million (95% UI: 56.5 to 83.6) in 2019 (37.3% increase), followed by an accelerated 25.7% surge to 85.6 million (95% UI: 70.3 to 103.8) during the pandemic years 2019–2021 (**Figure 1**, **Table 1**). This abrupt escalation contrasted sharply with the pre-pandemic annualized growth rate of 1.2%.

**Table 1:** Prevalence of major depressive disorders among WCBA in 1990, 2019, and 2021, and percentage change and estimated annual percentage change from 1990 to 2019 and 2019 to 2021.

Location	1990		2019		2021		1990 to 2019		2019 to 2021	
	No, in millions (95% UI)	Rates per 100 000 (95% UI)	No, in millions (95% UI)	Rates per 100 000 (95% UI)	No, in millions (95% UI)	Rates per 100 000 (95% UI)	PC in rates (100%)	EAPC in rates (95% CI)	PC in rates (100%)	EAPC in rates (95% CI)
Global	49.6 (41.6,60.2)	3711.99 (3107.64,4499.58)	68.1 (56.5,83.6)	3536.8 (2932.6,4339.14)	85.6 (70.3,103.8)	4394.55 (3607.71,5324.44)	−4.72	−0.38 (−0.48,−0.29)	24.25	11.47 (−0.56,24.95)
High-income Asia Pacific	1.1 (1,1.3)	2468.34 (2136.01,2856.96)	1 (0.9,1.2)	2574.7 (2226.47,3003.31)	1.2 (1,1.4)	3174.37 (2653.78,3775.21)	4.31	0.28 (0.07,0.49)	23.29	11.04 (6.65,15.6)
High-income North America	3.5 (2.9,4.1)	4648.8 (3948.14,5529.28)	5.2 (4.5,6.1)	6235.87 (5375.55,7297.17)	7.1 (6,8.2)	8403.17 (7163.83,9746.08)	34.14	0.67 (0.3,1.03)	34.76	16.08 (−1.83,37.27)
Western Europe	5.1 (4.5,6)	5379.78 (4704.47,6235.68)	5 (4.2,6)	5291.47 (4418.32,6382.18)	6.3 (5.2,7.8)	6714.94 (5552.91,8415.41)	−1.64	−0.03 (−0.07,0)	26.9	12.65 (−1.48,28.81)
Australasia	0.3 (0.3,0.4)	5941.22 (5049.7,7004.03)	0.4 (0.3,0.5)	6111.88 (4876.65,7560.89)	0.5 (0.4,0.6)	6716.15 (5132.76,8729.28)	2.87	0.2 (0.01,0.39)	9.89	4.83 (−1.3,11.33)
Andean Latin America	0.3 (0.2,0.4)	2951.71 (2365.98,3767.54)	0.5 (0.4,0.6)	2726.8 (2149.26,3492.26)	0.7 (0.5,0.9)	4095.73 (3094.97,5351.1)	−7.62	−0.38 (−0.43,−0.33)	50.2	22.56 (−9.61,66.17)
Tropical Latin America	2.1 (1.8,2.5)	5297.87 (4422.43,6329.98)	2.8 (2.4,3.3)	4698.81 (4021.51,5514.61)	3.9 (3.2,4.7)	6507.79 (5345.45,7815.24)	−11.31	−0.6 (−0.99,−0.21)	38.5	17.69 (8.18,28.03)
Central Latin America	1.3 (1.1,1.7)	3138.24 (2560.59,3939.01)	2.7 (2.2,3.3)	3956.85 (3228.17,4845.65)	3.5 (2.9,4.3)	5165.11 (4219.82,6339.22)	26.09	0.93 (0.87,0.99)	30.54	14.25 (−5.45,38.05)
Southern Latin America	0.6 (0.5,0.7)	4671.45 (3936.85,5758.35)	0.7 (0.6,0.8)	4096.46 (3463.44,4890.54)	0.9 (0.7,1.2)	5384.32 (4277.45,6754.92)	−12.31	−0.52 (−0.6,−0.44)	31.44	14.65 (−4.1,37.06)
Caribbean	0.5 (0.4,0.6)	4977.16 (4014.38,6187.3)	0.5 (0.4,0.6)	4092.12 (3194.57,5242.43)	0.6 (0.5,0.8)	5264.58 (4027.77,6974.08)	−17.78	−0.8 (−0.9,−0.7)	28.65	13.42 (3.92,23.8)
Central Europe	0.8 (0.6,1)	2588.88 (2099.6,3179.03)	0.6 (0.5,0.7)	2212.54 (1783.5,2734.21)	0.8 (0.6,1)	3107.93 (2472.71,3944.05)	−14.54	−0.85 (−0.98,−0.72)	40.47	18.52 (10.62,26.98)
Eastern Europe	1.9 (1.6,2.4)	3460.38 (2821.36,4261.1)	1.6 (1.3,2)	3324.07 (2640.24,4123.16)	2.3 (1.8,2.8)	4665.48 (3753.36,5776.95)	−3.94	−0.35 (−0.47,−0.22)	40.35	18.47 (15.53,21.49)
Central Asia	0.5 (0.4,0.6)	2863.77 (2302.91,3606.8)	0.7 (0.5,0.9)	2829.53 (2282.57,3593.9)	0.9 (0.7,1.1)	3590.23 (2826.46,4573.97)	−1.2	−0.02 (−0.06,0.03)	26.88	12.64 (4.22,21.75)
North Africa and Middle East	4.3 (3.5,5.4)	5504.45 (4479.99,6957.63)	8.6 (6.9,11)	5559.98 (4421.68,7068.68)	10.5 (8.2,13.4)	6618.58 (5161.96,8409.51)	1.01	0.1 (0.04,0.16)	19.04	9.11 (−3.61,23.5)
South Asia	11 (9,13.4)	4322.37 (3548.87,5241.68)	17.2 (14.2,20.9)	3579.32 (2958.01,4363.43)	22.7 (18.6,27.7)	4596.26 (3759.74,5609.98)	−17.19	−1.2 (−1.47,−0.92)	28.41	13.32 (−1.01,29.73)
Southeast Asia	2.3 (1.9,2.9)	1936.96 (1569.89,2424)	3.3 (2.7,4.1)	1812.23 (1470.06,2245.22)	4.3 (3.5,5.4)	2373.32 (1909.66,2971.05)	−6.44	−0.29 (−0.37,−0.21)	30.96	14.44 (13.49,15.4)
East Asia	9.1 (7.6,10.9)	2728.41 (2282.05,3278.26)	6.6 (5.5,8)	1907.6 (1593.24,2315.93)	6.1 (5,7.5)	1856.99 (1518.8,2265.64)	−30.08	−1.49 (−1.77,−1.21)	−2.65	−1.34 (−11.3,9.75)
Oceania	0 (0,0.1)	2506.84 (1972.04,3303.53)	0.1 (0.1,0.1)	2361.9 (1854.33,3058.76)	0.1 (0.1,0.1)	2591.77 (1933.05,3491.05)	−5.78	−0.24 (−0.27,−0.21)	9.73	4.75 (0.88,8.77)
Western Sub-Saharan Africa	1.6 (1.3,2)	3726.57 (3015.82,4693.83)	3.8 (3.1,4.8)	3412.47 (2723.45,4305.56)	4.4 (3.5,5.5)	3657.09 (2930.45,4619.54)	−8.43	−0.37 (−0.48,−0.26)	7.17	3.52 (−6.02,14.04)
Eastern Sub-Saharan Africa	1.9 (1.5,2.4)	4372.22 (3522,5538.86)	4 (3.2,5.1)	4005.54 (3218.91,5074.09)	5.2 (4.1,6.7)	4847.91 (3840.09,6246.09)	−8.39	−0.47 (−0.55,−0.39)	21.03	10.01 (4.94,15.33)
Central Sub-Saharan Africa	0.8 (0.6,1.1)	6687.09 (5158.5,8792.54)	1.9 (1.5,2.5)	6371.76 (4998.9,8179.35)	2.3 (1.8,3.1)	7122.05 (5471.94,9546.48)	−4.72	−0.17 (−0.19,−0.15)	11.78	5.72 (−5.52,18.3)
Southern Sub-Saharan Africa	0.5 (0.4,0.6)	4046.65 (3380.73,4878.35)	0.9 (0.7,1)	4076.84 (3377.07,4921.84)	1.2 (1,1.5)	5593.97 (4569.99,6945.23)	0.75	0.21 (0.05,0.38)	37.21	17.14 (6.56,28.76)
Low SDI	4.8 (3.9,6)	4285.53 (3448.12,5376.2)	10.1 (8.2,12.8)	3921.63 (3177.09,4968.65)	12.6 (10,16)	4593.77 (3661.11,5826.81)	−8.49	−0.53 (−0.65,−0.41)	17.14	8.23 (−0.5,17.73)
Low-middle SDI	11.7 (9.6,14.4)	4300.46 (3515.44,5263.48)	18.7 (15.4,23.1)	3812.77 (3131.27,4692.46)	24.2 (19.6,29.9)	4786.31 (3863.93,5911.09)	−11.34	−0.78 (−0.97,−0.59)	25.53	12.04 (−1.13,26.97)
Middle SDI	14.3 (11.9,17.5)	3203.76 (2662.51,3906.03)	18.4 (15.2,22.4)	2973.07 (2467.78,3627.15)	23 (18.9,27.8)	3711.02 (3058.73,4495.6)	−7.2	−0.42 (−0.52,−0.32)	24.82	11.72 (−0.61,25.58)
High-middle SDI	9.6 (8.1,11.4)	3438.44 (2899.03,4106.98)	9.6 (7.9,11.7)	3081.5 (2549.41,3744.66)	11.4 (9.2,14.1)	3739.77 (3021.35,4633.96)	−10.38	−0.53 (−0.66,−0.4)	21.36	10.16 (0.57,20.67)
High SDI	9.2 (8,10.8)	4059.95 (3525.55,4750.48)	11.2 (9.5,13.4)	4579.17 (3891.14,5476.41)	14.4 (12.2,17.1)	5915.76 (5023.98,7037.64)	12.79	0.3 (0.13,0.47)	29.19	13.66 (−0.82,30.26)

Prevalence rates per 100,000 population exhibited inverse trends. Between 1990 and 2019, rates declined moderately from 3,711.99 cases (95% UI: 3,107.64 to 4,499.58) to 3,536.8 cases per 100,000 population (95% UI, 2,932.6 to 4,339.14), yielding an estimated annual percentage change (EAPC) of −0.38 (95% CI: −0.48 to −0.29). However, the pandemic precipitated a dramatic reversal, with rates climbing to 4,394.55 (95% UI, 3,607.71 to 5,324.44) by 2021—equivalent to a 30.2-fold acceleration in annual growth rate (EAPC: 11.47; [95% CI: −0.56 to 24.95]) compared to the pre-pandemic period (**Figure 1**, **Table 1**).

This pandemic-driven pattern extended to incidence and disability-adjusted life years (DALYs), with all three metrics showing synchronized deviations from historical trajectories (**Supplementary Figures S1**, **S2**, **Supplementary Tables S1**, **S2**), indicating a systemic disruption of burden trajectories and an acute societal vulnerability.

### SDI and GBD regional trends

Regional disparities in MDDs burden among WCBA were analyzed through both SDI and geographic lenses. Across all metrics (prevalence, incidence, DALYs), three key patterns emerged: (1) absolute case concentration in middle- and low-middle regions, (2) accelerated pandemic-driven growth across all strata, and (3) divergent rate trajectories between SDI groups.

**Figure 2:**
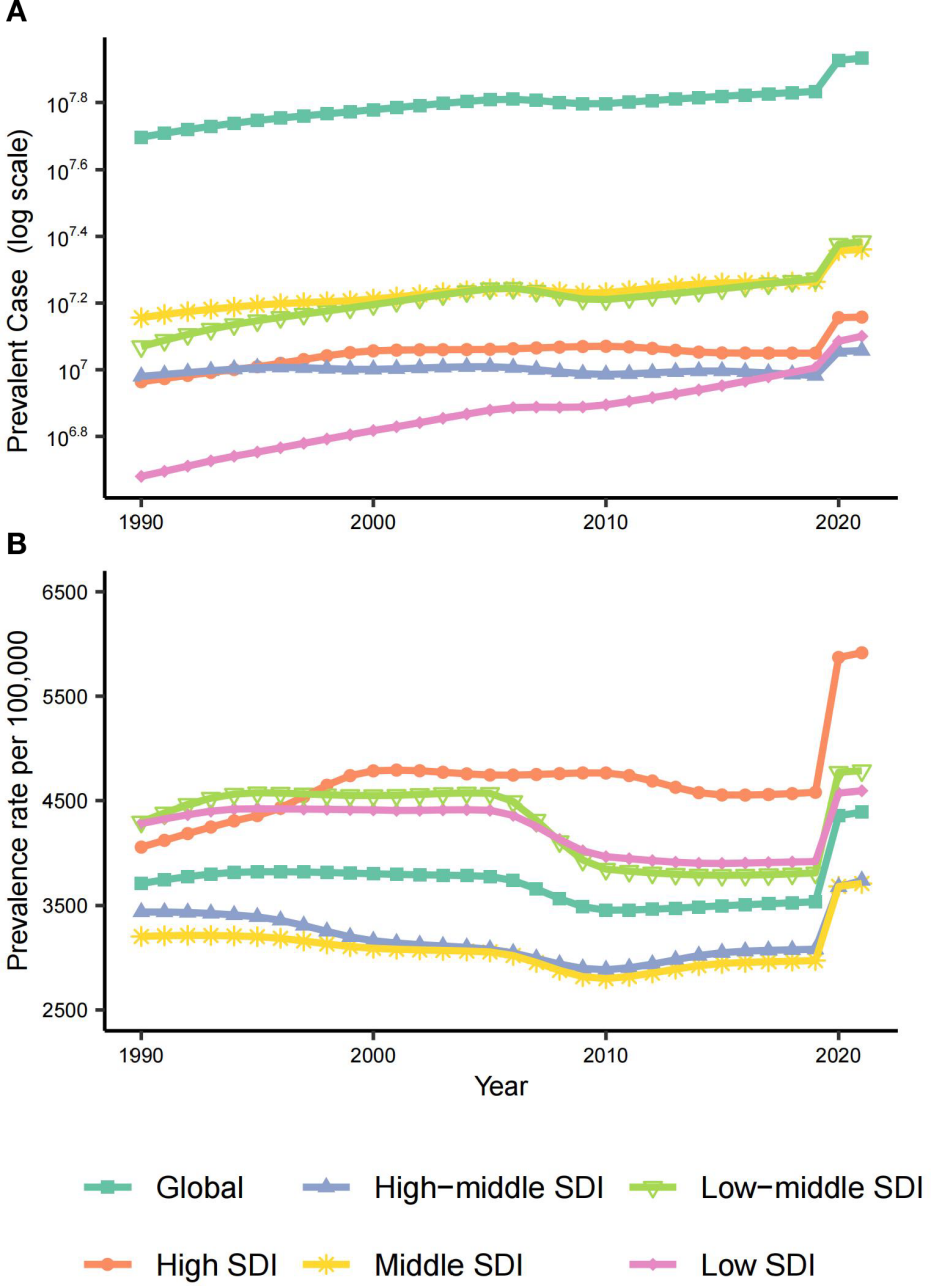
The global and 5 regions prevalence of MDD among WCBA from 1990 to 2021. **(A)** Prevalent case from 1990 to 2021. **(B)** Prevalence rates per 100,000 population from 1990 to 2021. WCBA, Women of Childbearing Age; MDD, major depressive disorder.

In 2021, middle- and low-middle regions carried the heaviest case burdens: Low-middle SDI regions accounted for 24.2 million cases (95% UI: 19.6 to 29.9; 28.3% global share) followed closely by Middle SDI regions with 23 million cases (95% UI: 18.9 to 27.8; 26.9% global share)—collectively representing 55.2% of global prevalent cases (**Figure 2A**, **Supplementary Figures S3**, **S4**, **Table 1**, **Supplementary Tables S1**, **S2**). However, Low SDI regions demonstrated the most dramatic case growth—a 160% increase from 1990 (4.8 million [95% UI: 3.9 to 6]) to 2021 (12.6 million [95% UI: 10 to 16]) (**Table 1**), outpacing population growth rates in these regions.

**Figure 3:**
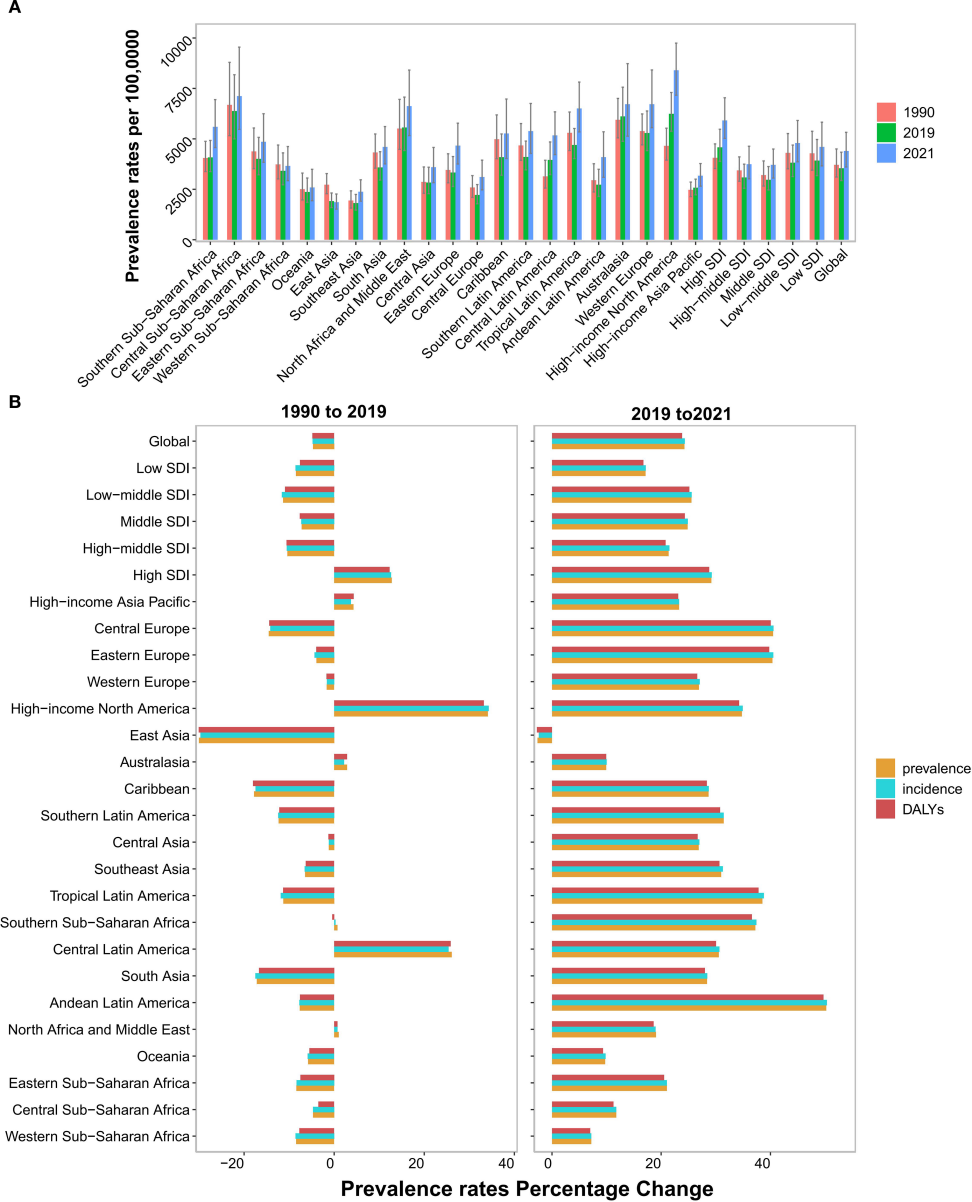
Temporal trend of MDD burden among WCBA in regions. **(A)** Prevalence rates per 100,000 population in 1990, 2019 and 2021. **(B)** The percentage change of prevalence, incidence and DALYs rates from 1990 to 2019, and 2019 to 2021. WCBA, Women of Childbearing Age; MDD, major depressive disorders; DALYs, disability-adjusted life years.

Prevalence rates revealed an inverse-U relationship with development levels. High SDI regions maintained persistently elevated rates, increasing from 4,059.95 (95% UI: 3,525.55 to 4,750.48) per 100,000 population in 1990 to 4,579.17 (95% UI: 3,891.14 to 5,476.41) in 2019, with a modest upward trend (EAPC: 0.3 [95% CI: 0.13 to 0.47]) (**Figure 3B**, **Table 1**). The pandemic triggered universal rate acceleration across SDI regions (2019–2021 EAPC range:8.23 to 13.66), most markedly in High SDI regions reaching 5,915.76 per 100,000 population (29.19% increase; EAPC:13.66 [−0.82 to 30.26])—though wide confidence intervals suggest pandemic-era volatility (**Figures 2**, **3**, **Table 1**). Intriguingly, SDI showed no linear correlation with prevalence rates (Spearman’s r=−0.07, p=0.07) (**Supplementary Figures S5**–**S7**). Middle and High-middle SDI regions paradoxically had the lowest prevalence rates, while Low-middle and Low SDI regions were closer to High SDI levels (**Figure 2**). This suggests complex mediation by factors like healthcare accessibility and cultural stigma beyond pure socioeconomic development.

Geographically, South Asia dominated absolute cases with 22.7 million (95% UI: 18.6 to 27.7) in 2021, followed by North Africa/Middle East and High-income North America (**Table 1**, **Supplementary Tables S1**, **S2**). High-income North America and Central Latin America exhibited significant pre-pandemic prevalence rate increases (**Figure 3B**, **Table 1**). Almost all geographical regions saw rising prevalence rates post-pandemic, with the largest increase in Andean Latin America (EAPC: 22.56 [95% CI: −9.61 to 66.17]). Notably, East Asia was the sole region with declining prevalence rates post-pandemic (EAPC: −1.34 [95% CI: −11.3 to 9.75]) (**Figure 3B**, **Table 1**). This contrasts with its pre-pandemic stability and may reflect sociocultural resilience. By 2021, High-income North America recorded the highest prevalence rate (8,403.17 [95% UI: 7,163.83 to 9,746.18]), while East Asia had the lowest (1,856.99 [95% UI: 1,518.8 to 2,265.64]) (**Figure 3A**).

### National trends

**Figure 4:**
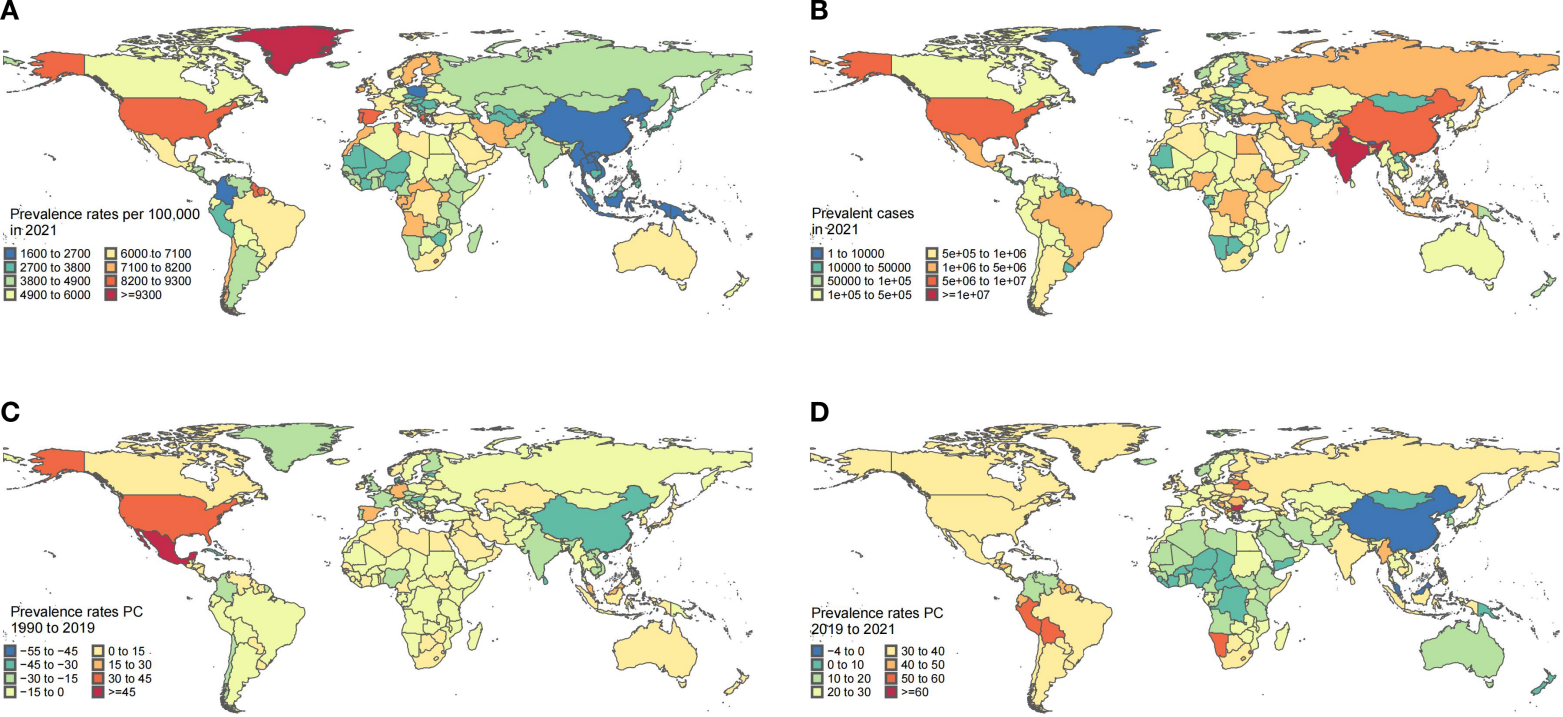
MDD burden among WCBA in nations. **(A)** Prevalence rates across 204 countries in 2021. **(B)** Prevalent case across 204 countries in 2021. **(C)** Percentage change in prevalence rates across 204 countries from 1990 and 2019. **(D)** Percentage change in prevalence rates across 204 countries from 2019 and 2021. WCBA, Women of Childbearing Age; MDD, major depressive disorders; PC, Percentage change.

Our analysis of 204 countries and territories revealed substantial heterogeneity in MDDs burden among WCBA. In 2021, India carried the highest absolute burden with 16.3 million prevalent cases (95% UI: 13.4 to 19.7), constituting 19% of the global total—more than the next America’s 6.6 million (95% UI: 5.6 to 7.6) and China’s 5.9 million (95% UI: 4.8 to 7.2) combined (**Figure 4B**, **Supplementary Tables S3–S5**). Strikingly, Greenland exhibited the highest prevalence rate (13,822.85 per 100,000 population [95% UI: 10,242.66 to 18,185.42]), potentially reflecting geographic isolation compounded by limited mental healthcare infrastructure (30, 31).

The pre-pandemic era (1990–2019) witnessed declining prevalence rates in 124 countries (60%), most notably Singapore (EAPC: −2.8 [95% CI: −3.13 to −2.47]), contrasting with Mexico’s significant increase (EAPC: 1.87 [95% CI: 1.64 to 2.1]) (**Figure 4C**, **Supplementary Tables S3**–**S5**). Post-pandemic (2019–2021), 99% of nations experienced escalation in MDDs burden among WCBA and Bulgaria showed the most dramatic prevalence surge (EAPC: 27.01 [95% CI: 24.54 to 29.53]). China (EAPC: −1.53 [95% CI: −11.81 to 9.95]) and Malaysia (EAPC: −1.04 [95% CI: −14.15 to 14.07]) emerged as outliers with declining prevalence rates (**Supplementary Tables S3**–**S5**), suggesting successful policy interventions.

### Age-specific burden patterns

**Figure 5:**
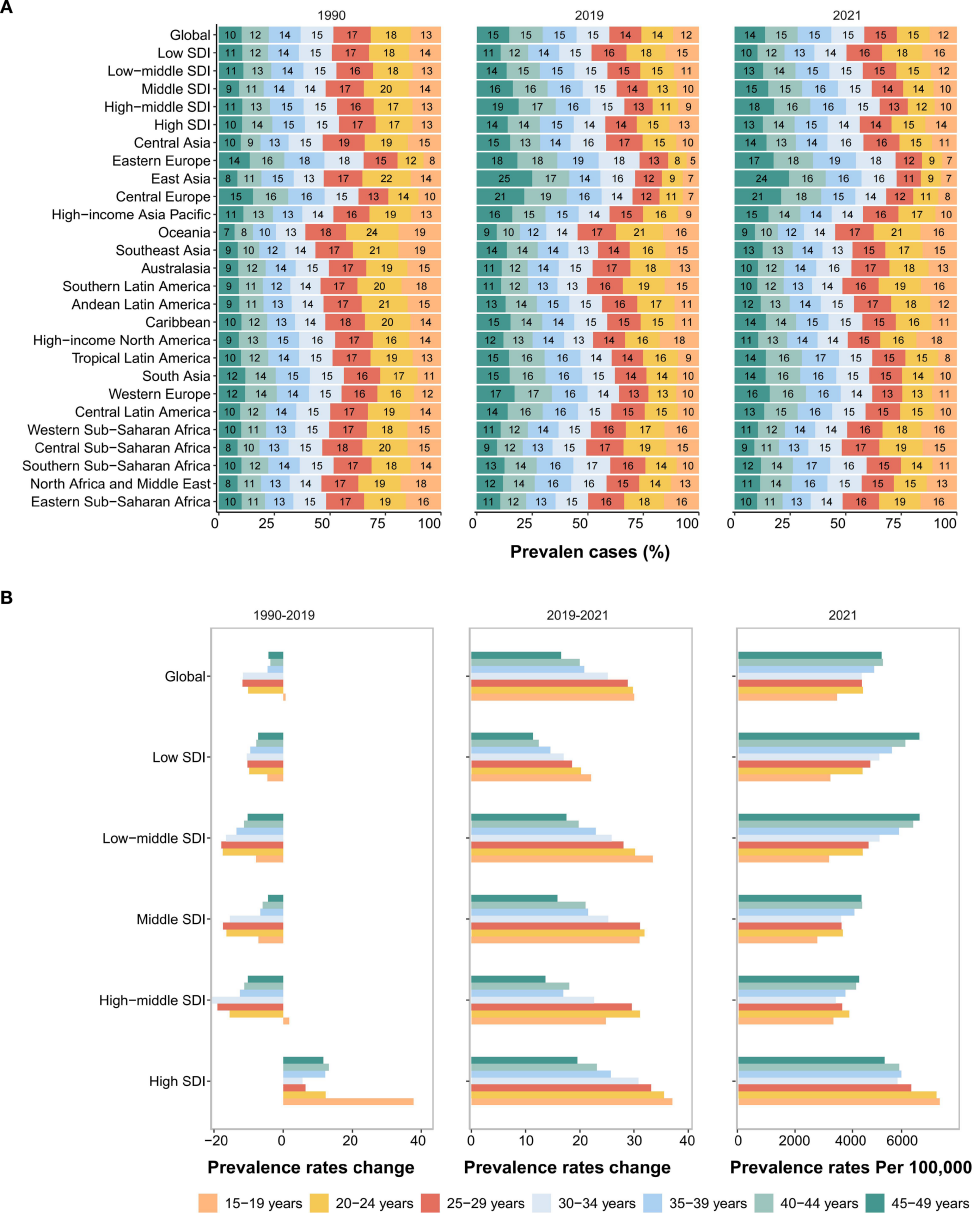
Temporal trend of MDD burden among WCBA by age pattern in different regions. **(A)** The distribution of prevalent numbers across 7 age groups as proportions globally, in 21 geographic regions and 5 SDI areas in 1990, 2019 and 2021. **(B)** Percentage change in prevalence rates of 7 age groups globally and regions from 1990 to 2019 (left panel) and from 2019 to 2021 (middle panel), and prevalence rates per 100,000 population in 2021 (right panel).

Our analysis revealed distinct age-related epidemiological patterns of MDDs among WCBA. During 1990 to 2019, while the global prevalence rates of most age groups experienced modest declines (average EAPC: −0.38 [95% CI: −0.48 to −0.29]), the 15–19 years cohort showed a paradoxical increase (EAPC: 0.04 [95% CI: −0.1 to 0.01]). This adolescent-specific pattern was most pronounced in High SDI regions, where prevalence rates surged by 37.7% (EAPC: 1.25 [95% CI: 1.05 to 1.45]) over three decades (**Figure 5B**, **Table 2**), potentially driven by unique societal stressors.

**Table 2:** Prevalence of major depressive disorders among WCBA in 1990, 2019, and 2021, and percentage change and estimated annual percentage change from 1990 to 2019 and 2019 to 2021.

Location	Age	1990		2019		2021		1990 to 2019		2019 to 2021	
		No, in millions (95% UI)	Rates per 100 000 (95% UI)	No, in millions (95% UI)	Rates per 100 000 (95% UI)	No, in millions (95% UI)	Rates per 100 000 (95% UI)	PC in rates (100%)	EAPC in rates (95% CI)	PC in rates (100%)	EAPC in rates (95% CI)
Global	15–49 years	49.6 (41.6,60.2)	3711.99 (3107.64,4499.58)	68.1 (56.5,83.6)	3536.8 (2932.6,4339.14)	85.6 (70.3,103.8)	4394.55 (3607.71,5324.44)	−4.72	−0.38 (−0.48,−0.29)	24.25	11.47 (−0.56,24.95)
Global	15–19 years	6.7 (4.7,9)	2613.25 (1827.83,3525.84)	7.8 (5.4,10.5)	2630.32 (1803.09,3534.45)	10.4 (7.1,13.9)	3420.94 (2328.34,4590.03)	0.65	−0.04 (−0.1,0.01)	30.06	14.04 (−0.74,31.03)
Global	20–24 years	9.1 (6.5,13)	3710.34 (2644.05,5332.62)	9.8 (6.7,14.6)	3334.76 (2282.82,4997.88)	12.7 (8.7,19)	4329.41 (2977.12,6466.42)	−10.12	−0.61 (−0.72,−0.49)	29.83	13.94 (−0.43,30.38)
Global	25–29 years	8.3 (6.4,11)	3769.52 (2896.53,4995.46)	9.8 (7.3,13.5)	3326.2 (2471.34,4567.68)	12.5 (9.4,17.1)	4285.94 (3214.92,5874.99)	−11.76	−0.65 (−0.76,−0.54)	28.85	13.51 (0,28.85)
Global	30–34 years	7.3 (5.4,9.8)	3865.53 (2825.98,5163.16)	10.1 (7.2,14.1)	3415.45 (2422.75,4738.58)	12.8 (9,17.5)	4273.99 (3021.78,5866.82)	−11.64	−0.59 (−0.66,−0.52)	25.14	11.86 (−0.33,25.55)
Global	35–39 years	7.1 (5.5,8.9)	4089.11 (3158.18,5102.43)	10.4 (7.8,13.2)	3904.97 (2935.12,4972.7)	13.1 (9.8,16.5)	4717.14 (3533.78,5945.7)	−4.5	−0.42 (−0.54,−0.31)	20.8	9.91 (−1.31,22.4)
Global	40–44 years	6.1 (4.6,7.9)	4341.26 (3282.8,5659.97)	10.2 (7.5,13.5)	4183.6 (3099.64,5560.21)	12.5 (9.1,16.3)	5018.67 (3667.69,6585.69)	−3.63	−0.4 (−0.53,−0.27)	19.96	9.53 (−0.44,20.5)
Global	45–49 years	5.1 (4.1,6.2)	4464.82 (3596.54,5468.45)	10 (8,12.3)	4273.44 (3422.08,5264.63)	11.7 (9.4,14.5)	4979.99 (3968.3,6172.29)	−4.29	−0.38 (−0.49,−0.28)	16.53	7.95 (−0.78,17.45)
Low SDI	15–49 years	4.8 (3.9,6)	4285.53 (3448.12,5376.2)	10.1 (8.2,12.8)	3921.63 (3177.09,4968.65)	12.6 (10,16)	4593.77 (3661.11,5826.81)	−8.49	−0.53 (−0.65,−0.41)	17.14	8.23 (−0.5,17.73)
Low SDI	15–19 years	0.7 (0.4,1)	2739.86 (1740.62,3882.09)	1.5 (1,2.2)	2614.17 (1656.11,3720)	2 (1.2,2.8)	3191.4 (2022.08,4499.62)	−4.59	−0.31 (−0.4,−0.22)	22.08	10.49 (−0.28,22.42)
Low SDI	20–24 years	0.9 (0.6,1.3)	3971.84 (2663.39,6110.21)	1.8 (1.2,2.7)	3582.48 (2332.57,5541.04)	2.3 (1.5,3.5)	4305.57 (2852.33,6644.69)	−9.8	−0.55 (−0.65,−0.45)	20.18	9.63 (−0.28,20.52)
Low SDI	25–29 years	0.8 (0.6,1.1)	4316.45 (3107.16,6080.01)	1.6 (1.1,2.3)	3870.25 (2760.78,5545.74)	2 (1.4,2.9)	4587.83 (3236.92,6506.33)	−10.34	−0.61 (−0.72,−0.49)	18.54	8.88 (−0.43,19.05)
Low SDI	30–34 years	0.7 (0.5,1)	4679.57 (3230.02,6530.26)	1.5 (1,2.1)	4186.9 (2857.96,5947.8)	1.8 (1.2,2.6)	4898.61 (3288.04,6884.49)	−10.53	−0.64 (−0.78,−0.5)	17	8.17 (−0.49,17.58)
Low SDI	35–39 years	0.7 (0.5,0.9)	5147.13 (3692.83,6807.84)	1.4 (1,1.9)	4658.4 (3351.31,6169.62)	1.7 (1.2,2.3)	5337.5 (3740.4,7089.99)	−9.5	−0.6 (−0.75,−0.45)	14.58	7.04 (−0.82,15.52)
Low SDI	40–44 years	0.6 (0.4,0.8)	5600.28 (3959.01,7707.08)	1.3 (0.9,1.7)	5165.6 (3658.46,7161.9)	1.5 (1.1,2.1)	5806.89 (4052.49,7947.94)	−7.76	−0.55 (−0.71,−0.39)	12.41	6.03 (−0.99,13.54)
Low SDI	45–49 years	0.5 (0.4,0.6)	6086.46 (4654.87,7650.66)	1.1 (0.8,1.4)	5647.65 (4342.1,7139.04)	1.3 (1,1.6)	6289.89 (4764.96,7912.69)	−7.21	−0.53 (−0.69,−0.37)	11.37	5.53 (−0.72,12.18)
Low-middle SDI	15–49 years	11.7 (9.6,14.4)	4300.46 (3515.44,5263.48)	18.7 (15.4,23.1)	3812.77 (3131.27,4692.46)	24.2 (19.6,29.9)	4786.31 (3863.93,5911.09)	−11.34	−0.78 (−0.97,−0.59)	25.53	12.04 (−1.13,26.97)
Low-middle SDI	15–19 years	1.5 (1,2.1)	2553.74 (1695.8,3557.56)	2.1 (1.4,2.9)	2353.83 (1584.28,3239.27)	2.8 (1.9,3.9)	3142.45 (2081.55,4297.56)	−7.83	−0.43 (−0.54,−0.32)	33.5	15.54 (−2,36.23)
Low-middle SDI	20–24 years	2.1 (1.5,3.1)	4012.21 (2833.75,5883.18)	2.8 (1.9,4.3)	3312.58 (2262.05,5027.63)	3.8 (2.6,5.7)	4313.23 (2941.81,6501.77)	−17.44	−0.96 (−1.13,−0.79)	30.21	14.11 (−2.02,32.89)
Low-middle SDI	25–29 years	1.9 (1.4,2.6)	4305.61 (3184.27,5870.26)	2.8 (2,3.9)	3534.18 (2592.98,4927.24)	3.7 (2.7,5.2)	4525.94 (3291.02,6422.07)	−17.92	−1.04 (−1.24,−0.84)	28.06	13.16 (−1.35,29.82)
Low-middle SDI	30–34 years	1.7 (1.2,2.4)	4670.79 (3291.01,6367.27)	2.8 (2,3.9)	3896.62 (2775.42,5379.92)	3.6 (2.5,5)	4905.44 (3384.42,6751.45)	−16.57	−1.04 (−1.27,−0.81)	25.89	12.2 (−0.81,26.92)
Low-middle SDI	35–39 years	1.7 (1.2,2.2)	5250.37 (3892.98,6755.94)	2.9 (2.1,3.8)	4542.03 (3346.11,5882.4)	3.7 (2.7,4.8)	5583.52 (4116.41,7176.87)	−13.49	−0.92 (−1.15,−0.7)	22.93	10.87 (−0.78,23.89)
Low-middle SDI	40–44 years	1.5 (1.1,2)	5719.94 (4161.05,7604.55)	2.8 (2,3.8)	5072.69 (3678.42,6841.11)	3.5 (2.5,4.7)	6074.33 (4343.94,8148.47)	−11.32	−0.86 (−1.07,−0.65)	19.75	9.43 (−0.97,20.92)
Low-middle SDI	45–49 years	1.3 (1,1.6)	5972.93 (4676.56,7441.67)	2.5 (2,3.1)	5358.14 (4226.22,6656.54)	3.1 (2.5,3.9)	6298.14 (4999.11,7801.95)	−10.29	−0.83 (−1.04,−0.62)	17.54	8.42 (−0.9,18.61)
Middle SDI	15–49 years	14.3 (11.9,17.5)	3203.76 (2662.51,3906.03)	18.4 (15.2,22.4)	2973.07 (2467.78,3627.15)	23 (18.9,27.8)	3711.02 (3058.73,4495.6)	−7.2	−0.42 (−0.52,−0.32)	24.82	11.72 (−0.61,25.58)
Middle SDI	15–19 years	2.1 (1.4,2.8)	2247.11 (1563.62,3068.65)	1.8 (1.2,2.4)	2085.67 (1436.75,2812.71)	2.4 (1.6,3.2)	2732.87 (1869.26,3698.19)	−7.18	−0.37 (−0.44,−0.3)	31.03	14.47 (−0.91,32.24)
Middle SDI	20–24 years	2.9 (2,4.2)	3287.63 (2320.27,4704.64)	2.4 (1.7,3.6)	2748.77 (1890.45,4034.51)	3.1 (2.2,4.7)	3626.15 (2517.19,5365.39)	−16.39	−0.79 (−0.91,−0.66)	31.92	14.86 (−0.56,32.66)
Middle SDI	25–29 years	2.5 (1.9,3.3)	3295.88 (2503.39,4382.98)	2.6 (1.9,3.5)	2723.35 (2033.32,3679.42)	3.2 (2.4,4.4)	3570.31 (2658.75,4827.43)	−17.37	−0.74 (−0.83,−0.65)	31.1	14.5 (0.39,30.59)
Middle SDI	30–34 years	2 (1.5,2.7)	3358.7 (2445.37,4486.21)	2.8 (2,3.9)	2843.09 (2031.35,3901.95)	3.5 (2.5,4.8)	3559.95 (2540.42,4846.69)	−15.35	−0.61 (−0.73,−0.5)	25.21	11.9 (−0.62,26)
Middle SDI	35–39 years	2 (1.5,2.5)	3544.89 (2703.28,4449.09)	2.9 (2.2,3.6)	3310.56 (2491.79,4185.41)	3.7 (2.8,4.6)	4023.71 (3015.05,5043.48)	−6.61	−0.49 (−0.65,−0.33)	21.54	10.25 (−1.35,23.2)
Middle SDI	40–44 years	1.6 (1.2,2.1)	3766.79 (2799.8,4988.2)	2.9 (2.1,3.8)	3543.61 (2620.48,4683.06)	3.5 (2.6,4.6)	4288.97 (3141.36,5642.98)	−5.92	−0.5 (−0.64,−0.35)	21.03	10.02 (−0.19,21.26)
Middle SDI	45–49 years	1.3 (1,1.6)	3849.77 (3073.37,4784.47)	3 (2.4,3.7)	3681.72 (2925.69,4539.52)	3.5 (2.8,4.3)	4265.57 (3396.77,5241.27)	−4.37	−0.39 (−0.5,−0.29)	15.86	7.64 (−1.25,17.33)
High-middle SDI	15–49 years	9.6 (8.1,11.4)	3438.44 (2899.03,4106.98)	9.6 (7.9,11.7)	3081.5 (2549.41,3744.66)	11.4 (9.2,14.1)	3739.77 (3021.35,4633.96)	−10.38	−0.53 (−0.66,−0.4)	21.36	10.16 (0.57,20.67)
High-middle SDI	15–19 years	1.2 (0.9,1.6)	2596.11 (1873.19,3442.25)	0.9 (0.6,1.2)	2640.6 (1811.28,3526.67)	1.1 (0.8,1.6)	3294.54 (2191.57,4552.22)	1.71	0.04 (−0.14,0.22)	24.76	11.7 (3.41,20.65)
High-middle SDI	20–24 years	1.7 (1.2,2.4)	3469.07 (2482.95,4898.55)	1.1 (0.7,1.6)	2934.31 (2002.17,4321.72)	1.4 (0.9,2)	3846.56 (2577.74,5709.09)	−15.42	−0.96 (−1.19,−0.73)	31.09	14.49 (4.06,25.97)
High-middle SDI	25–29 years	1.6 (1.2,2)	3427.93 (2655.13,4447.59)	1.2 (0.9,1.7)	2777.29 (2095.89,3786.05)	1.5 (1.1,2)	3600.24 (2655.47,4944.14)	−18.98	−0.93 (−1.04,−0.81)	29.63	13.86 (3.32,25.46)
High-middle SDI	30–34 years	1.4 (1.1,1.9)	3457.29 (2573.02,4574.86)	1.5 (1,2)	2749.67 (1966.3,3767.98)	1.7 (1.2,2.4)	3371.6 (2343.97,4660.58)	−20.47	−0.73 (−0.84,−0.62)	22.62	10.73 (0.64,21.84)
High-middle SDI	35–39 years	1.4 (1.1,1.8)	3631.61 (2837.96,4503.47)	1.5 (1.1,1.9)	3178.72 (2376.82,4055.34)	1.8 (1.4,2.3)	3716.24 (2749.76,4730.17)	−12.47	−0.58 (−0.76,−0.4)	16.91	8.12 (−2.07,19.38)
High-middle SDI	40–44 years	1.2 (0.9,1.6)	3900.77 (2916.52,5069.78)	1.6 (1.2,2.1)	3460.37 (2539.56,4634.43)	1.9 (1.4,2.5)	4085.26 (2970.57,5454.98)	−11.29	−0.63 (−0.78,−0.47)	18.06	8.65 (−0.83,19.04)
High-middle SDI	45–49 years	1 (0.8,1.2)	4101.98 (3314.18,5024.74)	1.8 (1.5,2.2)	3683.46 (2944.64,4519.63)	2 (1.6,2.5)	4186.63 (3243,5240.21)	−10.2	−0.56 (−0.66,−0.47)	13.66	6.61 (−1.64,15.55)
High SDI	15–49 years	9.2 (8,10.8)	4059.95 (3525.55,4750.48)	11.2 (9.5,13.4)	4579.17 (3891.14,5476.41)	14.4 (12.2,17.1)	5915.76 (5023.98,7037.64)	12.79	0.3 (0.13,0.47)	29.19	13.66 (−0.82,30.26)
High SDI	15–19 years	1.2 (0.9,1.5)	3708.27 (2714.89,4801.49)	1.5 (1.1,1.9)	5106.4 (3759.16,6476.48)	2 (1.5,2.6)	7000.04 (5189.32,8909.44)	37.7	1.25 (1.05,1.45)	37.08	17.08 (−0.9,38.33)
High SDI	20–24 years	1.5 (1.1,2.1)	4530.5 (3358.31,6302.81)	1.6 (1.2,2.3)	5085.28 (3619.41,7284.28)	2.2 (1.6,3.1)	6892.67 (4984.08,9875.55)	12.25	0.23 (0.12,0.34)	35.54	16.42 (−0.37,36.04)
High SDI	25–29 years	1.5 (1.2,1.9)	4242.62 (3408.66,5334.15)	1.6 (1.3,2.1)	4515.24 (3520.19,5893.45)	2.1 (1.6,2.7)	6013.86 (4632.2,7718.77)	6.43	−0.1 (−0.24,0.05)	33.19	15.41 (−1.24,34.86)
High SDI	30–34 years	1.4 (1.1,1.8)	4007.47 (3058.94,5160.09)	1.6 (1.2,2.1)	4230.23 (3100.14,5624.68)	2.1 (1.5,2.8)	5534.16 (4040.65,7366.23)	5.56	−0.13 (−0.31,0.05)	30.82	14.38 (−1.18,32.38)
High SDI	35–39 years	1.3 (1.1,1.6)	4014.6 (3314.31,4852.1)	1.7 (1.4,2.1)	4501.05 (3592.39,5622.61)	2.1 (1.7,2.7)	5660.85 (4525.34,7106.42)	12.12	0.23 (0.01,0.44)	25.77	12.15 (−1.18,27.27)
High SDI	40–44 years	1.3 (1,1.6)	4009.66 (3219.45,4979.86)	1.6 (1.2,2.1)	4537.24 (3499.54,5798.62)	2.1 (1.6,2.6)	5586.97 (4301.43,7125.73)	13.16	0.43 (0.2,0.66)	23.14	10.97 (−0.39,23.61)
High SDI	45–49 years	1 (0.8,1.2)	3814.94 (3194.04,4572.46)	1.6 (1.3,1.9)	4256.23 (3454,5199.33)	1.8 (1.5,2.2)	5088.47 (4141.69,6160.97)	11.57	0.42 (0.23,0.61)	19.55	9.34 (−0.46,20.1)

The COVID-19 pandemic amplified existing age disparities. Between 2019 to 2021, all age groups exhibited significant prevalence rate increases, with magnitude inversely correlated with age. Adolescents (15–19 years) experienced the steepest rise by 30.06% (EAPC: 14.04 [95% CI: −0.74 to 31.03]), nearly double the average growth observed in older cohort (45–49 years) (**Figure 5B**, **Table 2**). Strikingly, East Asia demonstrated exceptional resilience, achieving an 11.53% (EAPC: −5.94 [95% CI: −13.87 to 2.71]) reduction in adolescent prevalence rates—a phenomenon potentially attributable to rapid implementation of school-based mental health interventions (**Figure 5B**, **Supplementary Table S6**).

By 2021, the global burden distribution revealed two critical peaks: Case magnitude in 20–44 years groups and Prevalence intensity in 40–49 years groups (**Figures 5A, B**, **Supplementary Table S6**). Notably, the global 15–19 years cohort maintained the lowest baseline prevalence rate (3420.94 per 100,000 population [95% UI: 2,328.34 to 4,590.03])—a pattern unchanged since 1990—but highest pandemic-driven growth (**Figure 5B**, **Supplementary Table S6**). This contrarian trend (low baseline rates vs. rapid pandemic surge) highlights adolescent vulnerability to acute societal disruptions. This pattern persisted across SDI strata except high-SDI regions, where prevalence rates remained elevated (≥7,000 per 100,000 population) in adolescent ages (15–19 years) (**Figure 5B**, **Supplementary Table S6**).

### Long-term projections and pandemic legacy

The pandemic-driven surge in MDDs burden among WCBA may have long-term implications. Using ARIMA models (auto.arima() in R), we projected prevalence trends under two scenarios: Pre-pandemic baseline (1990–2019 data) and Pandemic-inclusive (1990–2021 data). The Ljung-Box test confirmed that the model residuals were white noise (**Supplementary Table S9**).

**Figure 6:**
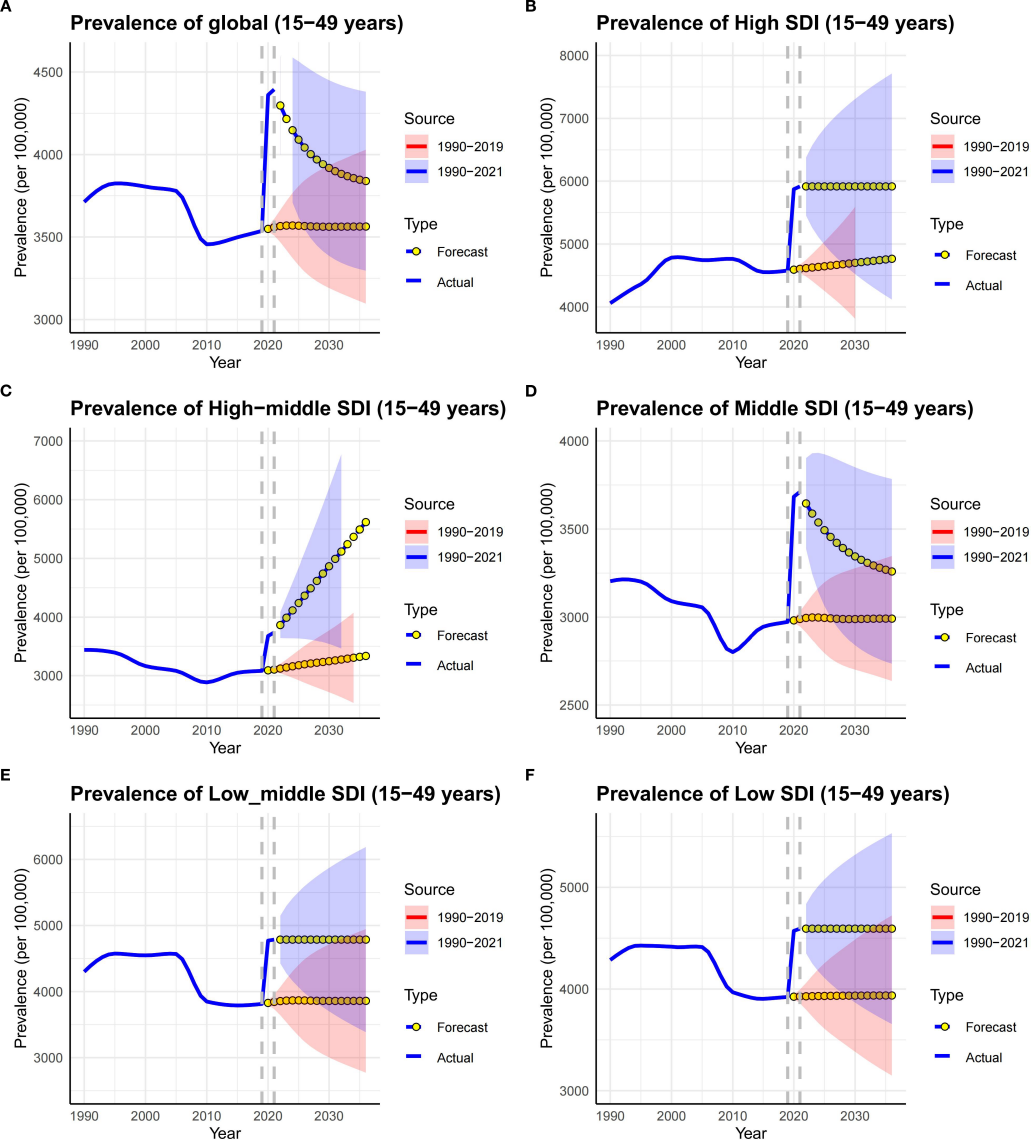
Time trends of prevalence in MDD among WCBA in SDI regions and globally from 1990 to 2036. Solid lines represent the actual trend, blue dot lines and shaded regions (Red represent predictions based on data from 1990 to 2021, and Blue represent predictions based on data from 1990 to 2019) represent the forecasted trend and its 95% CI. **(A–F)** respectively represent the trends in the Global, High SDI, High-middle SDI, Middle SDI, Low-middle SDI, and Low-SDI regions.

Pandemic-inclusive projections predict 103.06 million global prevalent cases by 2036, 32% higher than pre-pandemic estimates (78.21 million) (**Supplementary Table S10**). Global prevalence rate may decline to 3,838.43 per 100,000 population by 2036 but remain 7.7% above pre-pandemic projections (3,563.11 per 100,000 population) (**Figure 6A**, **Supplementary Table S10**), suggesting incomplete post-crisis recovery. The same trend can also be observed in Middle SDI regions.

However, in High SDI, Low-middle SDI and Low SDI regions, the prevalence rates are likely to remain high for the next 15 years (**Figures 6B, E, F**, **Supplementary Table S10**). High SDI regions are predicted to sustain the highest prevalence rates (5617.68 per 100,000 population in 2036), exceeding 2019 levels by 22.7% (**Figure 6B**, **Supplementary Table S10**) and reflecting persistent societal stressors. Low-SDI regions face dual challenges: prevalent cases projected to grow by 30% (2021–2036); prevalence rates persisting at 4593.77 per 100,000 population in 2036.

Age-specific projections of global prevalence rates reveal divergent trends. Pandemic-inclusive projections show an accelerated decline in the 20–24, 35–39 and 40–44 age groups, but remain higher than pre-pandemic projections. On the other hand, the 25–29, 30–34, and 45–49 age groups are likely to persistent at high levels. The 45–49 age group is projected to maintain peak rate (4979.99 per 100,000 population) by 2036. Notably, the 15–19 age group is projected to rapidly decrease to pre-pandemic levels (**Supplementary Figure S8**, **Supplementary Table S10**).​​ These projections indicate the COVID-19 pandemic’s dual legacy: transient global rate surges and long-term age-specific vulnerabilities, except among adolescent women.

## Discussion

The COVID-19 pandemic profoundly reshaped global MDD burden among WCBA, exacerbating pre-existing vulnerabilities while introducing novel societal stressors. Unlike earlier analyses aggregating all depressive disorders (32), this study focuses on MDD among WCBA. We reveal a dual challenge: High SDI regions face persistently elevated prevalence rates (5,915.76 per 100,000 population in 2021), driven by systemic stressors such as workplace competition (33, 34) and heightened psychological vulnerability in post-material societies (35, 36), or by medical transparency such as advanced medical systems and diagnosis and treatment capabilities (36, 37); while Low SDI regions grapple with rapid case expansion (160% growth since 1990) fueled by population dynamics (38), healthcare inequities (39), and socioeconomic instability (40–42). Notably, the pandemic reversed pre-2019 stability: Global cases surged 25.7% (2019–2021), disproportionately affecting adolescents (15–19 years: +30.06% prevalence rate), underscoring acute societal disruptions. This acceleration aligns with global reports of pandemic-induced mental health declines (21), yet our age- and region-specific analysis uncovers critical nuances. For instance, East Asia’s unique decline in adolescent prevalence rate (−2.65% post-2019) may reflect sociocultural resilience (e.g., family support systems) and targeted policy interventions (43–46), contrasting sharply with global trends. These disparities underscore the complex interplay of biological susceptibility (e.g., hormonal fluctuations in adolescence and perimenopause (47–49), structural inequities, and pandemic-driven disruptions, necessitating tailored strategies to address this escalating public health crisis.

The age-specific disparities in MDD burden among WCBA further illuminate the pandemic’s differential impact across developmental stages. Adolescents (15–19 years) exhibited the sharpest post-2019 surge globally (30.06% increase in prevalence rate), likely exacerbated by pandemic-related disruptions such as social isolation, academic stress, future uncertainty, and familial stress (50–52). This aligns with neurodevelopmental vulnerabilities during adolescence, where hormonal fluctuations and incomplete prefrontal cortex maturation heighten sensitivity to environmental stressors (52, 53). Strikingly, East Asia defied this trend with an 11.53% decline in adolescent prevalence, potentially attributable to the epidemic’s short-term effects (such as academic decompression), regional policy interventions (such as preferential treatment of mental health), and sociocultural resilience (such as family support). However, more longitudinal studies are needed to analyze the underlying reasons for its deviation from global trends. Conversely, the 40–49 age groups maintained the highest global prevalence rate (about 5,000 per 100,000 population in 2021), underscoring the interplay of perimenopausal hormonal shifts and cumulative life stressors (e.g., caregiving roles, occupational burnout) (4, 48). These findings reveal a pandemic-driven amplification of age-specific vulnerabilities. For instance, the 45–49 group’s projected persistence of elevated rates through 2036 suggests that acute societal disruptions may entrench long-term mental health inequities, particularly in regions lacking targeted interventions. On the contrary, the 15–19 age group’s prevalence rates are predicted to return to pre-pandemic levels by 2036, suggesting that adolescent depressive symptoms during the pandemic may primarily reflect acute stress responses rather than entrenched psychopathology. Addressing these disparities demands life-course approaches, such as integrating hormonal health into adolescent mental health programs and expanding perimenopausal care access in primary healthcare systems.

The stark regional disparities in MDD burden among WCBA underscore the complex interplay of socioeconomic development, healthcare infrastructure, and cultural contexts. High SDI regions, such as High-income North America, exhibited the highest prevalence rates (8,403.17 per 100,000 population in 2021), reflecting a paradoxical burden where advanced healthcare systems improve diagnostic transparency but fail to mitigate stressors like workplace competition, social isolation, and amplify spiritual needs (33–37). Conversely, Low SDI regions experienced the fastest case growth (160% since 1990), driven by population expansion and systemic inequities such as limited mental health resources and economic instability (38, 40–42). These regions likely face underreporting due to diagnostic biases and stigma, suggesting the true burden may far exceed current estimates. Middle SDI regions, while reporting the lowest prevalence rates (3,711.02 per 100,000 population in 2021), paradoxically bear the relatively high absolute caseloads (23 million in 2021), emphasizing the need to address population-scale risks masked by moderate rates. The unique resilience observed in East Asia—particularly its adolescent prevalence decline (−11.53% post-2019)—may stem from synergistic policy actions (e.g., rapid integration of mental health into primary care) and cultural norms prioritizing collective well-being over individual stressors (43, 45). However, the projected persistence of elevated rates in high SDI regions (5,617.68 per 100,000 population by 2036) signals entrenched structural vulnerabilities, such as gendered caregiving roles amplified by pandemic-related remote work. To bridge these gaps, interventions must be stratified: high SDI settings require workplace reforms and digital mental health innovations (54, 55), while low SDI regions demand grassroots screening programs and economic empowerment initiatives targeting WCBA.

The COVID-19 pandemic’s enduring impact on MDD burden among WCBA extends beyond acute infection risks, embedding long-term mechanisms through socioeconomic, healthcare, and psychosocial disruptions. First, prolonged healthcare interruptions—such as reduced access to perinatal mental health services and contraceptive care—exacerbated pre-existing vulnerabilities, particularly in Low SDI regions where maternal health infrastructure was already fragile (10, 42). Second, economic precarity, intensified by job losses and inflationary pressures, disproportionately affected women in informal labor sectors, amplifying financial stress and caregiving burdens (33, 40). This aligns with studies showing that income inequality and unemployment rates correlate strongly with MDD incidence in crises (34, 56). Third, the erosion of social support networks—through school closures, remote work conflicts, and restricted community gatherings—disproportionately strained WCBA, who often juggle dual roles as caregivers and income earners (51, 52). These disruptions may have epigenetic implications: chronic stress during the pandemic could alter hypothalamic-pituitary-adrenal (HPA) axis regulation, potentially entrenching depressive susceptibility across generations (47, 49). Notably, ARIMA projections suggest these effects are not transient; even if prevalence rates stabilize, the absolute caseload will rise by 32% by 2036, reflecting population growth compounded by pandemic-induced mental health scarring. Regionally, High SDI regions face persistent stressors (e.g., remote work blurring work-life boundaries (36), while Low SDI regions struggle with delayed healthcare recovery and intergenerational poverty cycles (38, 39). Mitigating this legacy demands systemic reforms: integrating mental health into universal healthcare coverage, expanding paid parental leave policies, and deploying community-based resilience programs to buffer future shocks. While our ARIMA model captures structural breaks induced by the pandemic, future projections would benefit from incorporating more recovery-phase data to refine attenuation parameters, particularly regarding adolescents’ resilience thresholds.

### Limitation

While this study provides critical insights into the evolving burden of MDD among WCBA, several limitations warrant consideration. First, GBD tool assumes consistent disease progression across populations and this may not fully capture cultural variations in symptom reporting; Reliance on GBD estimates introduces potential biases, as underreporting in Low SDI regions—due to stigma, diagnostic gaps, and fragmented health systems—likely underestimates the true burden, necessitating ground-truthing through community-based surveys. Second, the ARIMA model’s projections, though robust for short-term trends, may inadequately capture long-term societal shifts (e.g., post-pandemic economic recovery or mental health policy reforms) that could alter trajectory patterns. For instance, the model assumes continuity of pandemic-induced stressors but does not account for emerging interventions such as digital mental health platforms or universal basic income trials, which may mitigate future burdens. Third, the short observation window for pandemic effects (2019–2021) limits our ability to disentangle transient shocks from sustained trends, particularly in regions like East Asia where early declines may reflect temporary policy buffers rather than durable resilience. Future research should prioritize longitudinal studies to track age-specific vulnerabilities (e.g., adolescent cohorts into adulthood) and integrate mixed-methods approaches to explore cultural mediators of mental health outcomes, such as familial support networks in East Asia or stigma dynamics in Low SDI regions. Addressing these gaps will strengthen the evidence base for equitable, context-driven interventions in the post-pandemic era.

## Conclusion

The COVID-19 pandemic has intensified MDD burden among WCBA, exposing stark disparities: High SDI regions exhibit elevated prevalence rates, while Low SDI regions face rapid case growth. Adolescents experienced the sharpest burden surge, yet East Asia defied trends with a decline, underscoring sociocultural resilience and policy efficacy. Persistent vulnerabilities demand stratified interventions: digital mental health tools in High SDI regions, grassroots screening in Low SDI regions, and adolescent-focused programs globally. Urgent action is needed to address this dual crisis, combining equitable healthcare access and gender-sensitive reforms to mitigate long-term mental health burdens.

## Data Availability

The datasets presented in this study can be found in online repositories. The names of the repository/repositories and accession number(s) can be found below: http://ghdx.healthdata.org/gbd-results-tool.
