# Supplementary material for: Burden and trends of major depressive disorders among women of childbearing age and the impact of the COVID-19 pandemic: insights from the global burden of disease study 2021
Source: Front Psychiatry. 2025 Sep 18;16:1630601. doi: 10.3389/fpsyt.2025.1630601 (PMC12489947; doi:10.3389/fpsyt.2025.1630601)
Supplement: Supplementary file 1 [file SupplementaryFile1.zip › Supplementary Figure 5.PDF]

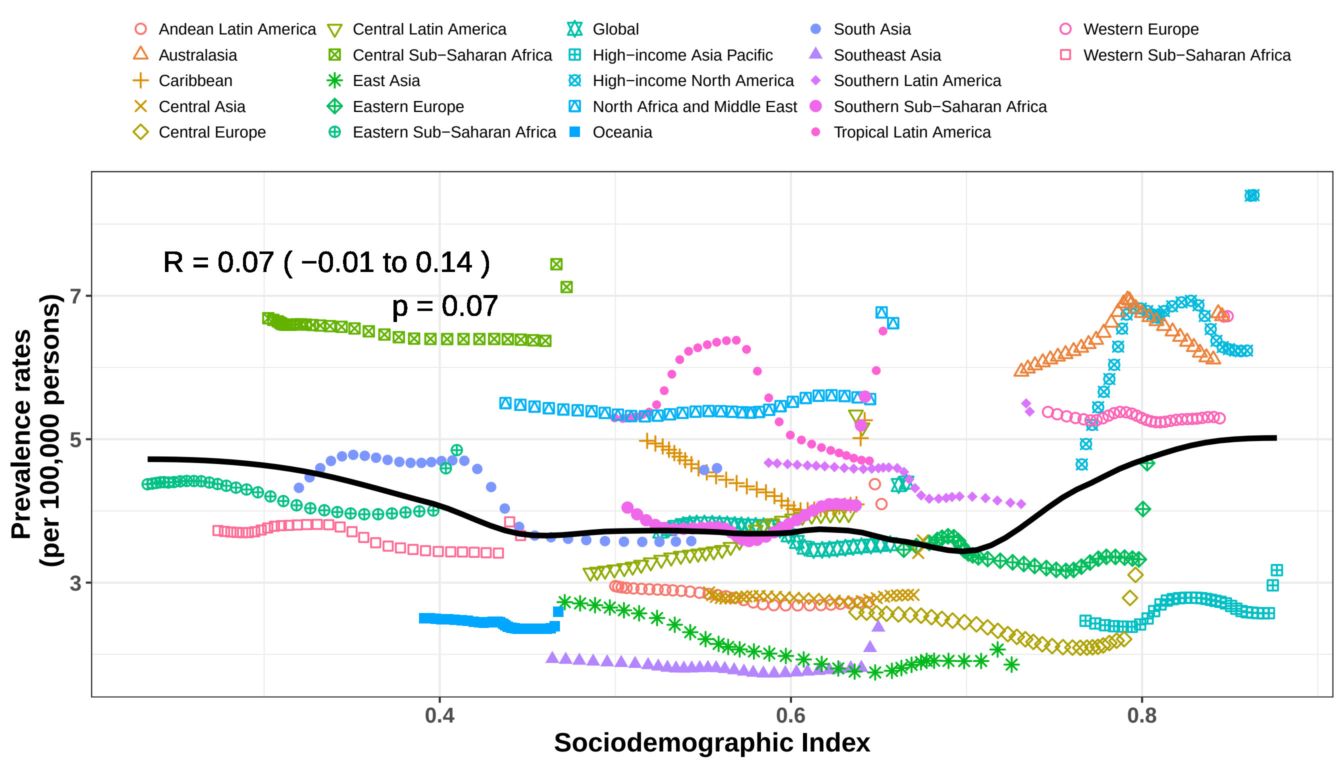

Andean Latin America
Australasia
Caribbean
Central Asia
Central Europe
Central Latin America
Central Sub−Saharan Africa
East Asia
Eastern Europe
Eastern Sub−Saharan Africa
Global
High−income Asia Pacific
High−income North America
North Africa and Middle East
Oceania
South Asia
Southeast Asia
Southern Latin America
Southern Sub−Saharan Africa
Tropical Latin America
Western Europe
Western Sub−Saharan Africa
R = 0.07 (−0.01 to 0.14)
p = 0.07
Prevalence rates
(per 100,000 persons)
7
5
3
0.4
0.6
0.8
Sociodemographic Index
