# Supplementary material for: Burden and trends of major depressive disorders among women of childbearing age and the impact of the COVID-19 pandemic: insights from the global burden of disease study 2021
Source: Front Psychiatry. 2025 Sep 18;16:1630601. doi: 10.3389/fpsyt.2025.1630601 (PMC12489947; doi:10.3389/fpsyt.2025.1630601)
Supplement: Supplementary file 1 [file SupplementaryFile1.zip › Supplementary Figure Captions.DOCX]

**Supplementary Figure Legends**

**Supplementary Figure 1. The incidence of major DD among WCBA from 1990 to 2021**. The bar graph shows the incidence numbers (in millions) from 1990 to 2021 on the left-hand y-axis, while the line graph represents the incidence rates per 100,000 over the same period on the right-hand y-axis. WCBA: Women of Childbearing Age; DD: depressive disorder

.

**Supplementary Figure 2. The DALYs of major DD among WCBA from 1990 to 2021.**

The bar graph shows the DALYs numbers (in millions) from 1990 to 2021 on the left-hand y-axis, while the line graph represents the DALYs rates per 100,000 over the same period on the right-hand y-axis. WCBA: Women of Childbearing Age; DD: depressive disorder

**Supplementary Figure 3. The global and 5 regions incidence of major DD among WCBA from 1990 to 2021**.

**A** Incidence numbers from 1990 to 2021. **B** Incidence rates per 100,000 population from 1990 to 2021. WCBA: Women of Childbearing Age; DD: depressive disorder;

**Supplementary Figure 4. The global and 5 regions DALYs of major DD among WCBA from 1990 to 2021**.

**A** DALYs numbers from 1990 to 2021. **B** DALYs rates per 100,000 population from 1990 to 2021. WCBA: Women of Childbearing Age; DD: depressive disorder;

**Supplementary Figure 5. The associations between the SDI and prevalent rates per 100,000 population of major DD among WCBA across 21 GBD regions.**

**Supplementary Figure 6. The associations between the SDI and incidence rates per 100,000 population of major DD among WCBA across 21 GBD regions.**

**Supplementary Figure 7. The associations between the SDI and DALYs rates per 100,000 population of major DD among WCBA across 21 GBD regions.**

**Supplementary Figure 8. Time trends of prevalence in major DD among WCBA in age groups from 1990 to 2036.**

Solid lines represent the actual trend, blue dot lines and shaded regions (Red represent predictions based on data from 1990 to 2021, and Blue represent predictions based on data from 1990 to 2019) represent the forecasted trend and its 95% CI.
