# Supplementary figures and images for: Burden and trends of major depressive disorders among women of childbearing age and the impact of the COVID-19 pandemic: insights from the global burden of disease study 2021
Source: Front Psychiatry. 2025 Sep 18;16:1630601. doi: 10.3389/fpsyt.2025.1630601 (PMC12489947; doi:10.3389/fpsyt.2025.1630601)

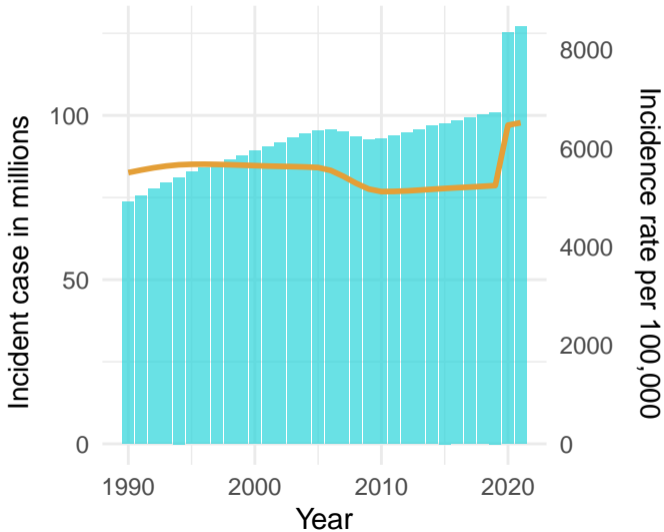

Supplement: Supplementary file 1 [file SupplementaryFile1.zip › Supplementary Figure 1.PDF]

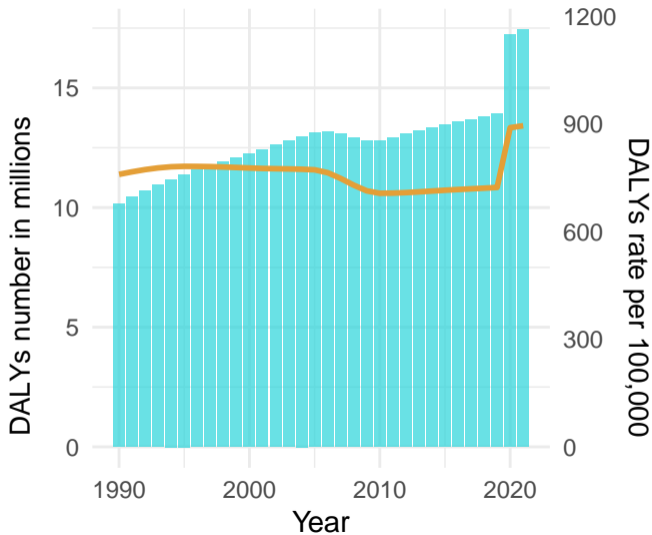

Supplement: Supplementary file 1 [file SupplementaryFile1.zip › Supplementary Figure 2.PDF]

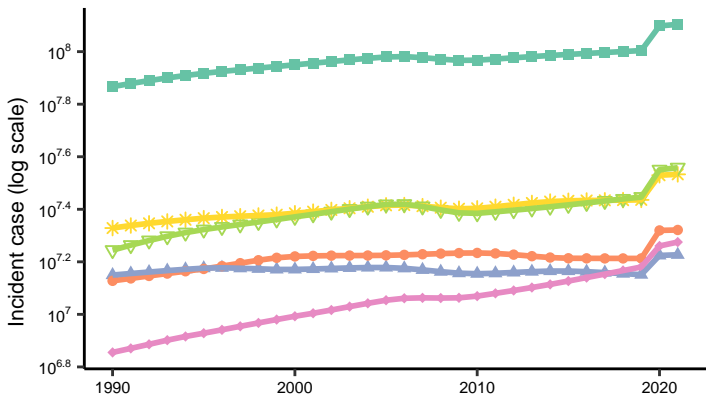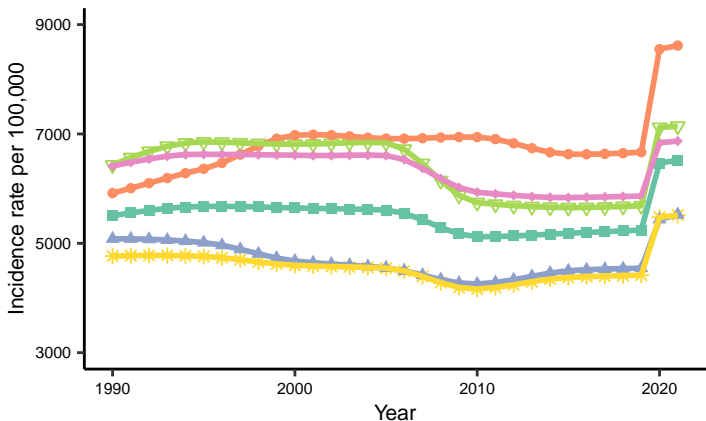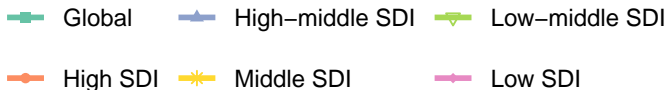

Supplement: Supplementary file 1 [file SupplementaryFile1.zip › Supplementary Figure 3.PDF]

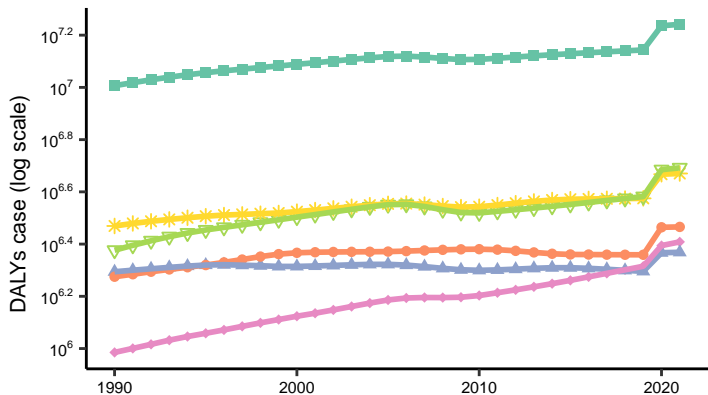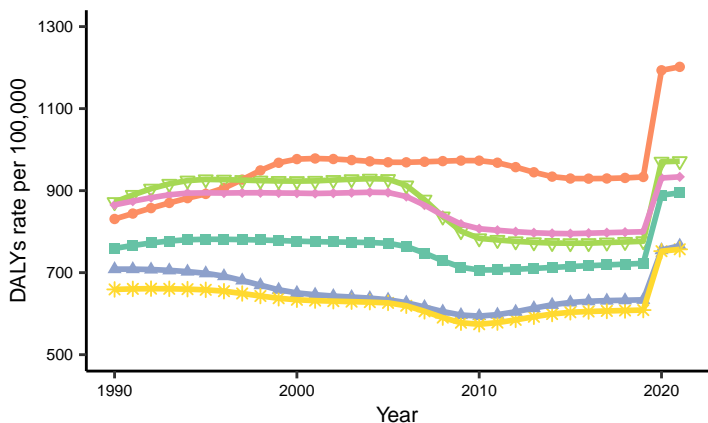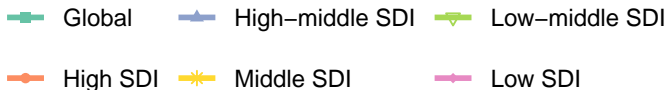

Supplement: Supplementary file 1 [file SupplementaryFile1.zip › Supplementary Figure 4.PDF]

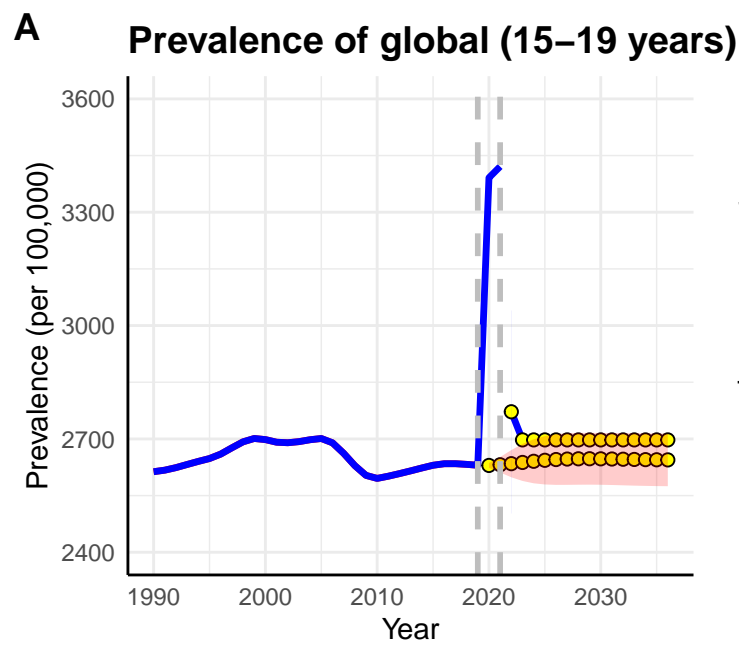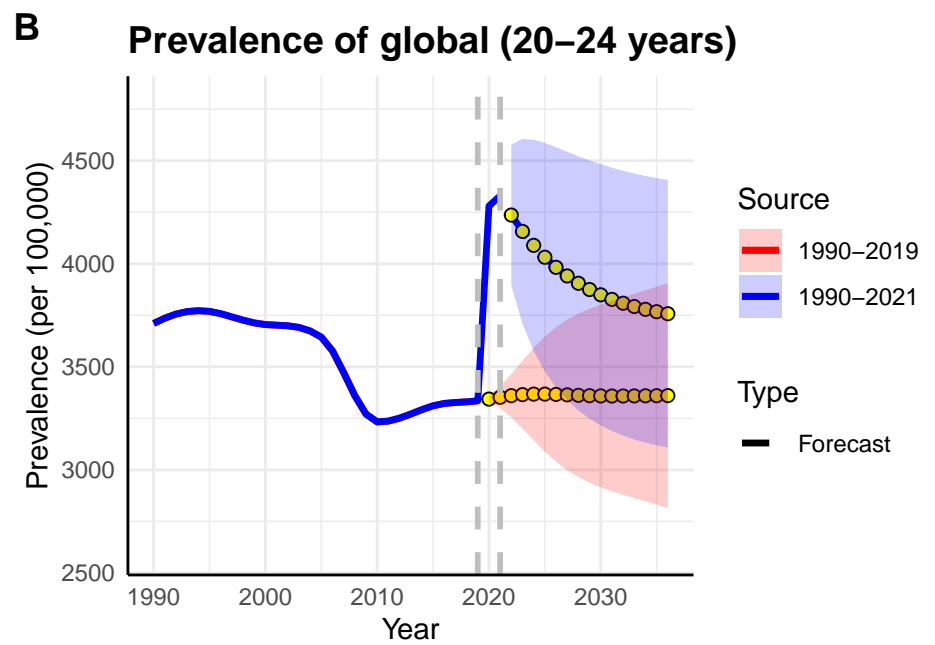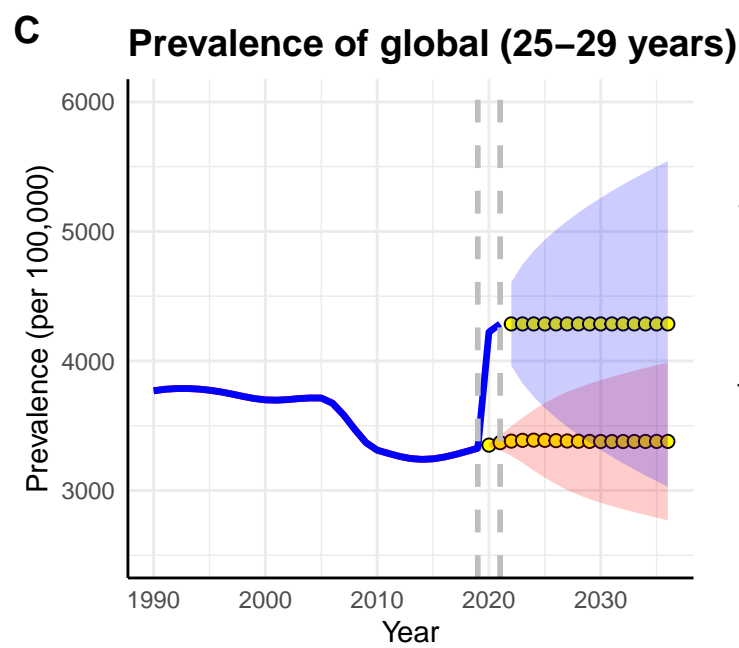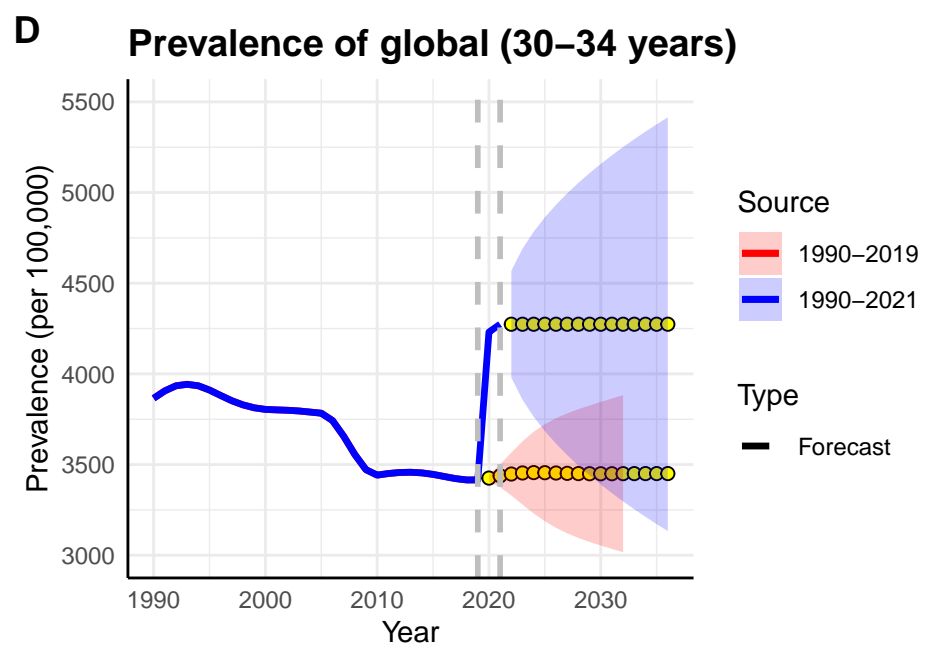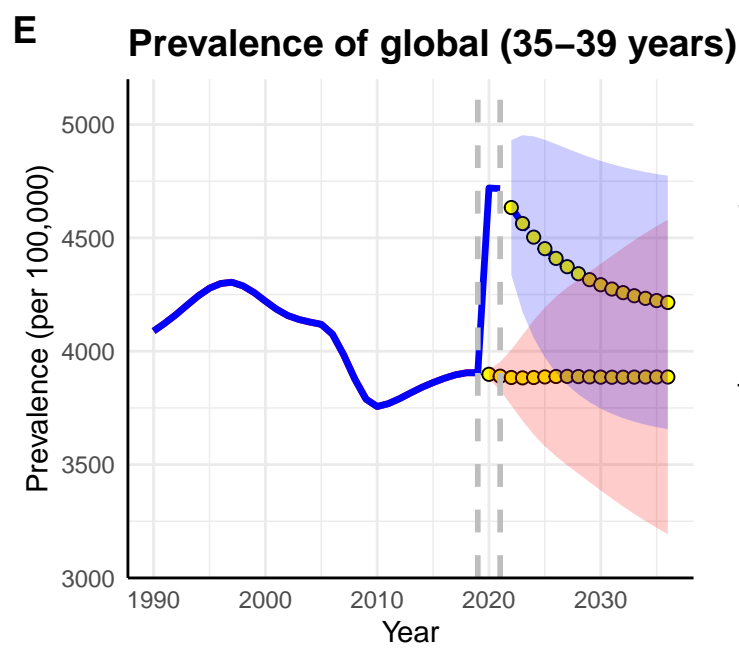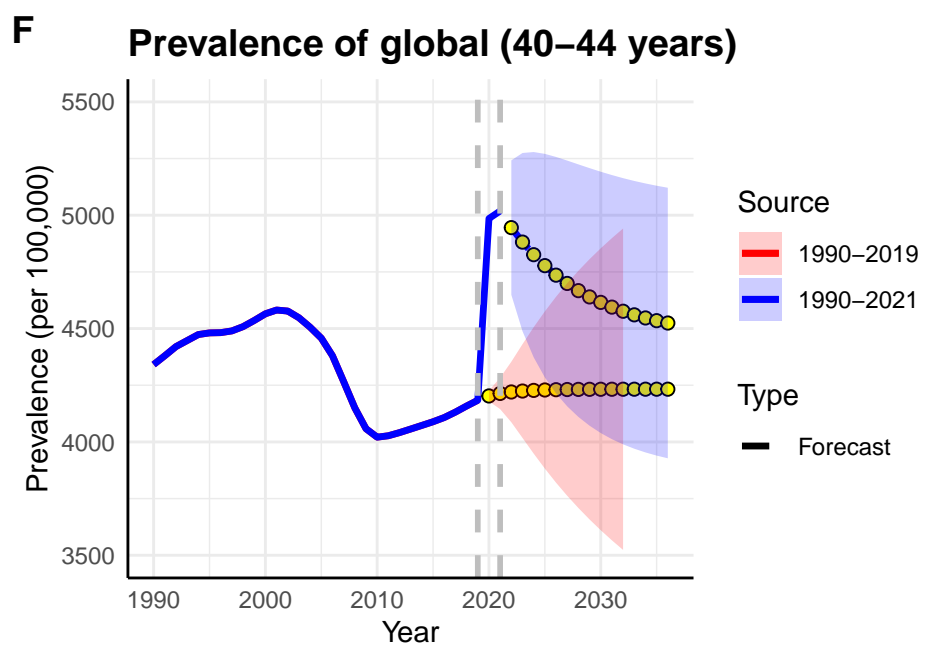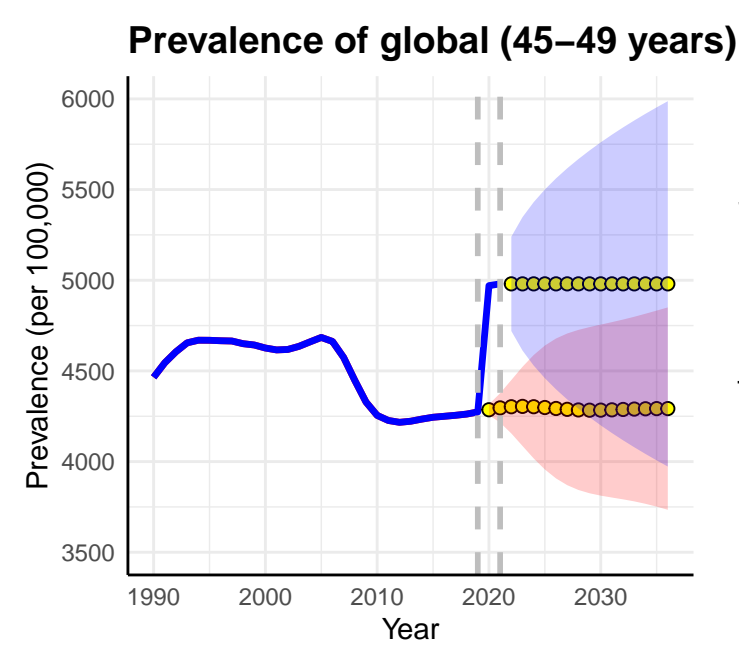

Supplement: Supplementary file 1 [file SupplementaryFile1.zip › Supplementary Figure 8.PDF]
